# Potential mechanism of Luoshi Neiyi prescription in endometriosis based on serum pharmacochemistry and network pharmacology

**DOI:** 10.3389/fphar.2024.1395160

**Published:** 2024-07-29

**Authors:** Lizheng Wu, Shuhong Lin, Yongjun Hu, Shangwen Jing, Bowen Sun, Xiaoxin Chen, Jinjin Jia, Cheng Zeng, Fangli Pei

**Affiliations:** ^1^ Guangzhou University of Chinese Medicine, Guangzhou, Guangdong, China; ^2^ Department of Gynecology, The First Affiliated Hospital of Guangzhou University of Chinese Medicine, Guangzhou, Guangdong, China

**Keywords:** endometriosis, Luoshi Neiyi prescription, traditional Chinese medicine, HIF1A/EZH2/ANTXR2 pathway, network pharmacology, molecular docking, molecular dynamics simulation

## Abstract

**Introduction:**

Endometriosis (EMs) is characterized by ectopic growth of active endometrial tissue outside the uterus. The Luoshi Neiyi prescription (LSNYP) has been extensively used for treating EMs in China. However, data on the active chemical components of LSNYP are insufficient, and its pharmacological mechanism in EMs treatment remains unclear. This study aimed to explore the potential mechanism of LSNYP for EMs through network pharmacology based on the components absorbed into the blood.

**Methods:**

Ultra-high performance liquid chromatography-quadrupole time-of-flight mass spectrometry was used to analyze blood components, and a series of network pharmacology strategies were utilized to predict targets of these components and EMs. Protein–protein interaction (PPI) network analysis, component–target–disease network construction, gene ontology (GO) functional enrichment analysis, and Kyoto Encyclopedia of Genes and Genomes (KEGG) pathway enrichment analysis were performed. Additionally, molecular docking, molecular dynamics simulations, and *in vitro* and *in vivo* experiments were conducted to validate the HIF1A/EZH2/ANTXR2 pathway associated with hypoxic pathology in EMs.

**Results:**

Thirty-four absorbed components suitable for network pharmacology analysis were identified, and core targets, such as interleukin 6, EGFR, HIF1A, and EZH2, were founded. Enrichment results indicated that treatment of EMs with LSNYP may involve the regulation of hypoxia and inflammatory-related signaling pathways and response to oxidative stress and transcription factor activity. Experimental results demonstrated that LSNYP could decrease the expression of HIF1A, ANTXR2, YAP1, CD44, and β-catenin, and increased EZH2 expression in ectopic endometrial stromal cells and endometriotic tissues. Molecular docking and molecular dynamics simulations manifested that there was stable combinatorial activity between core components and key targets of the HIF1A/EZH2/ANTXR2 pathway.

**Conclusion:**

LSNYP may exert pharmacological effects on EMs via the HIF1A/EZH2/ANTXR2 pathway; hence, it is a natural herb-related therapy for EMs.

## 1 Introduction

Endometriosis (EMs) is a chronic inflammatory disease characterized by the ectopic growth of active endometrial tissue (glands and stroma) beyond the confines of the uterus, affecting approximately 10% of females of childbearing age (Shafrir et al., 2018). EMs is the primary etiological factor for dysmenorrhea, chronic pelvic pain, and infertility, imposing a substantial burden on patients’ quality of life and social healthcare resources (Zubrzycka et al., 2021). In contemporary medical practice, therapeutic interventions, such as non-steroidal anti-inflammatory drugs, compound oral contraceptives, progesterone, gonadotropin-releasing hormone agonists, and surgical procedures are commonly employed to manage the condition. Traditional Chinese medicine (TCM) teaches that cyclic bleeding from the ectopic endothelium is indicative of stasis, and that the core pathogenesis of EMs is the stagnation of qi and blood. Consequently, the treatment approach often involves promoting qi and eliminating blood stasis to dissipate stagnation. The Luoshi Neiyi prescription (LSNYP), documented in Luo Yuankai’s gynecology book, has been extensively used in the First Affiliated Hospital of Guangzhou University of Chinese Medicine and Guangzhou Women and Children’s Medical Center for EMs treatment based on this fundamental principle (Ya-Nan et al., 2020; Kefei et al., 2022). LSNYP have demonstrated efficacy in suppressing endometriotic lesions, alleviating chronic pain, and reducing recurrence rates in clinical settings (Haiying et al., 2013; Ye et al., 2021). Furthermore, experimental studies have indicated that LSNYP induces atrophy of the ectopic endometrium and reduces cyst size (Liao et al., 2010; Guo, 2021). Nevertheless, data on the active chemical components of LSNYP are insufficient, and its pharmacological mechanism in EMs treatment remains unclear.

Network pharmacology is an interdisciplinary field rooted in systems biology, elucidating the mechanisms of drug action and investigating the interrelationships between drugs and diseases by constructing a comprehensive “component–target–pathway–disease” network (Wu et al., 2022). This approach is particularly relevant for understanding the intricate nature of TCM, which emphasizes a multi-component, multi-pathway, and multi-target approach for treatment (Luo et al., 2019). Research in serum pharmacochemistry suggests that drug components can only exert their intended effects in the body once they are absorbed into the bloodstream via the digestive tract and reach their target organs or targets (Ma et al., 2017). However, traditional network pharmacology predominantly relies on databases to identify TCM components, thereby lacking assurance regarding the efficacy of the selected components. Hence, building on the prior identification of the chemical components of LSNYP *in vitro*, this study subsequently analyzed its prototype components entering the blood and explored the potential mechanism of action of LSNYP to provide a scientific basis for its actual utilization.

## 2 Materials and methods

### 2.1 Reagents

LSNYP (batch number: Yue Yao zhi Z20071247), with each bottle containing 250 mL, were produced by Guangzhou Kangyuan Pharmaceutical Co., Ltd. The production process was as follows: 14 types of Chinese medicines (Table 1) were mixed, and the mixture was firstly immersed in 2,500 mL of water for 30 min. The mixture was subsequently heated until boiling, decocted gently for 1.5 h, and filtered. The drug residue was boiled in 1,250 mL of water, decocted gently for 1 h, and filtered again. The two extracts were then mixed and concentrated to 250 mL, supplemented with 0.125 g of ethylp-hydroxybenzoate and 0.75 g of sodium benzoate, and stored in bottles.

**TABLE 1:** Fourteen botanical drugs in 250 mL of LSNYP.

Chinese name	Name for publishing	Used parts	Amount (g)
Yimucao (YMC)	*Leonurus japonicus* Houtt. [Lamiaceae; Herba Leonuri]	Aboveground parts	33.5
Chuanxiong (CX)	*Conioselinum anthriscoides “Chuanxiong”* [umbelliferae; *Chuanxiong Rhizoma*]	Tuber	10.25
Taoren (TR)	*Prunus persica* (L.) Batsch [Rosaceae; *Persicae Semen*]	Seed	17
Danshen (DS)	*Salvia miltiorrhiza* Bunge [Lamiaceae; *Salviae Miltiorrhizae Radix Et Rhizoma*]	Root	17
Puhuang (PH)	*Typha angustifolia* L. [Typhaceae; *Typhae Pollen*]	Pollen	6.5
Yanhusuo (YHS)	*Corydalis yanhusuo* (Y.H.Chou & Chun C.Hsu) W.T.Wang ex Z.Y.Su & C.Y.Wu [Papaveraceae; *Corydalis Rhizoma*]	Tuber	17
Wuyao (WY)	*Lindera aggregata* (Sims) Kosterm. [Lauraceae; *Linderae Radix*]	Root	17
Haizao (HZ)	*Sargassum pallidum* (Turn.) C.Ag. [Sargassaceae; *Sargassum*]	Whole plant	33.5
Zhebeimu (ZBM)	*Fritillaria thunbergii* Miq. [Liliaceae; *Fritillariae Thunbergii Bulbus*]	Bulb	17
Wumei (WM)	*Prunus mume* (Siebold) Siebold & Zucc. [Rosaceae; *Mume Fructus*]	Fruit	11.25
Tubiechong (TBC)	*Eupolyphaga sinensis* Walker [Corydidae; *Eupolyphaga Steleophaga*]	Whole body	13.5
Shanzha (SZ)	*Crataegus pinnatifida* Bunge [Rosaceae; *Crataegi Fructus*]	Fruit	17
Wulingzhi (WLZ)	*Trogopterus xanthipes* Milne-Edwards [Talpidae; *Faeces Trogopterori*]	Dung	11.25
Muli (ML)	*Ostrea gigas* Thunberg [Ostreidae; *Ostreae Concha*]	Shell	28.25

Penicillin sodium for injection (North China Pharmaceutical Group Semisyntech Co., Ltd., No. QM2201201) and danazol capsules (Jiangsu Lianhuan Pharmaceutical Co., Ltd., No. 20221201) were purchased from the Pharmacy of the First Affiliated Hospital of Guangzhou University of Chinese Medicine. Estradiol benzoate injection (batch number: 210301) was purchased from Shanghai Full Woo Biotechnology (Zhumadian) Company, and avertin (EasyCheck, 2104A) was purchased from Nanjing Aibei Biotechnology Co., Ltd. The following reagents, cell counting kit-8 (CCK-8; APExBIO, K1018), fetal bovine serum (FBS; Gibco, 10099141C), 0.25% trypsin + phenol red (Gibco, 25200056), type I collagenase (Sigma, C0130), anti-HIF1A antibody (Affinity, BF8002), anti-EZH2 antibody (Proteintech, 21800-1-AP), anti-ANTXR2 antibody (Solarbio, K004586P), anti-YAP1 antibody (Proteintech, 66900-1-Ig), anti-CD44 antibody (Proteintech, 15675-1-AP), anti-beta catenin antibody (Proteintech, 51067-2-AP), and anti-GAPDH antibody (Goodhere biotech, AB-P-R 001) were purchased with the help of Guangzhou Huiwang Experimental Instrument Co., Ltd. Trizol (Ambion, 15596-026), HiScript^®^ II Q Select RT SuperMix for qPCR(VAZYME, R233), SYBR Green Master Mix (VAZYME, Q111-02), Taq Plus DNA Polymerase (TIANGEN, ET105-01), DL2000 DNA Marker (TIANGEN, MD114-02), TIANamp Genomic DNA Kit (TIANGEN, DP304), EpiTect Plus DNA Bisulfite Kit (QIAGEN, 59124), TransTaq DNA Polymerase High Fidelity (HiFi) (TransGen, AP131-02), and HIF1A downregulated lentiviral vector were obtained from Wuhan Bio-Tower Biotechnology Co., Ltd.

### 2.2 Animals

Seventy-two female specific pathogen-free Sprague–Dawley rats, aged approximately 10–12 weeks, were purchased from the Experimental Animal Center of Guangzhou University of Chinese Medicine (Certificate of Quality No: SCXK Yue 2018-0034) and fed at the Clean Experimental Animal Center of the First Affiliated Hospital of Guangzhou University of Chinese Medicine. The housing conditions were as follows: a temperature of 23°C ± 2°C, humidity of 40%–70%, a 12-h light-dark cycle, and free access to food and water. The experimental ethics were reviewed and approved by the First Affiliated Hospital of Guangzhou University of TCM Animal Ethical Review Standards (approval number: TCMF1-2021024).

### 2.3 Preparing *in vivo* serum sample of LSNYP

After 3 days of adaptive feeding, before the start of drug administration, twelve rats were fasted for 12 h. Rats were randomly divided into four groups: the control group and administration groups 1, 2, and 3, with three rats per group. As the dose of LSNYP administered to a 60 kg woman is 75 mL/d, according to the body surface area conversion method (Guanhua, 2014), a 200 g rat is equivalent to 0.018 times that of a 70 kg adult. Therefore, the equivalent dose for a rat is 0.78 mL/100 g, equivalent to 7.82 g/kg/d of the original dose. As the dosage of each drug in the prescription was small, considering the metabolic differences and the relatively low serum drug concentration after gavage with an equivalent drug dose in the preliminary experiment, the administration groups were orally administered a quadruple dose of LSNYP. This dosage was divided into two doses, one in the morning and one in the evening, for three consecutive days. Rats in the control group were administered an equal volume of normal saline. To comprehensively analyze these components, blood samples were collected from the abdominal aortas of rats in the treatment groups at 0.5, 1.5, and 4 h (three rats each time) after the last gavage, and blood was collected from the control group at 1.5 h after saline administration. Blood samples were stored at 25°C for 2 h and then centrifuged at 3,000 rpm for 10 min to separate the serum. Finally, the sera of three rats in each group were mixed and preserved.

Equal volumes of serum from the three treatment groups were mixed, and methanol (chromatographic grade) was added at a 1:2 (serum: methanol) volume ratio. The mixture was vortexed for 5 min and then centrifuged at 13,000 rpm for 15 min at 4°C. The supernatant was dried in a centrifugal vacuum concentrator, and the residues were redissolved in 300 μL of methanol, vortexed for 5 min, and centrifuged at 13,000 rpm for 15 min. The supernatant was used for analysis. Serum samples from the control group were similarly processed.

An additional thirty rats were divided into control, LSNYP, and positive drug (danazol) groups. The control and LSNYP groups were subjected to the same procedure, as described above. The rats in the positive drug group were administered danazol (8.4 g/kg/d), a synthetic androgen with anti-estrogenic effects, dissolved in an equal volume of normal saline. All rats were anesthetized 1 h after the last gavage, and blood samples were collected from the abdominal aorta. The samples were allowed to clot at 25°C and centrifuged at 3,000 rpm for 10 min. The serum was subsequently inactivated in a water bath at 56°C for 30 min. Following filtration and sterilization, using a 0.22 μm filter membrane, the serum was stored at −80°C for subsequent cell experiments.

### 2.4 Ultra-high performance liquid chromatography-quadrupole time-of-flight mass spectrometry (UPLC-Q/TOF-MS) analysis

Chromatographic separation was performed using a Waters ACQUITY UHPLC system (Waters Corporation, Massachusetts, United States) with a Waters ACQUITY UPLC HSS T3 column (100 mm × 2.1 mm, 1.8 μm). The mobile phase comprised acetonitrile (A) and water containing 0.1% formic acid (B) with a gradient elution of 4% A at 0–3 min, 4%–43% A at 3–26 min, 43%–70% A at 26–32 min, and 70%–100% A at 32–35 min. The flow rate was set at 0.35 mL/min, and the injection volume was 2 μL.

A Waters Xevo G2 Q-T high-resolution mass spectrometer (MS) equipped with an electrospray ion source was used in this study. Data were collected in positive and negative ion modes, with capillary voltages set at 2.5 kV (+) and 2.5 kV (−), cone voltage at 40 V (−40 V), ion source temperature at 110°C, desolvation gas (N_2_) temperature at 550°C, desolvation gas flow at 900 L/h, cone gas flow at 50 L/h, and scanning range from 50 to 1,300 m/z. Leucine enkephalin was used as the internal standard for real-time quality correction. Data were collected using a fast data-dependent acquisition method, and secondary ions corresponding to the top 10 primary mass spectra were gathered in each cycle. The collision energies for high and low molecular weights were 55 and 25 V, respectively.

By reviewing the pertinent literature and databases (Liu et al., 2022b), a summary of the chemical components of each botanical drug in LSNYP was compiled, including their Chinese and English names, molecular formulas, and structural formulas. A specialized database encompassing 517 components was established. The raw data were initially analyzed using UNIFI 1.8 software. The parameter settings were as follows: a peak intensity threshold was 200 counts for high energy and 1,000 counts for low energy in the 3D peak detection; component identity match tolerance set to five parts-per-million (ppm), and fragment match tolerance set to 20 ppm. The final results were confirmed through manual analysis.

### 2.5 Network pharmacology research methods

#### 2.5.1 Screening the drug-likeness of absorbed components

Drug-likeness screening of compounds is a common method used to assess their absorption, distribution, metabolism, and excretion properties, often employing Lipinski’s Rule of Five (Lipinski et al., 2012). In 2012, Bickerton et al. (2012) proposed a new drug-likeness descriptor called the Quantitative Estimate of Drug-Likeness (QED). Weight-based QED can be used to better evaluate the drug-likeness of compounds. The intersection of the previously mentioned absorbed prototype components and the chemical components with the Isomeric Simplified Molecular Input Line Entry System structure in the PubChem database was obtained, and ADMET lab2.0 (Guoli et al., 2021), an integrated online platform for accurate and comprehensive predictions of ADMET properties (https://admetmesh.scbdd.com), was used to screen the intersection components. To obtain the final potentially active components of LSNYP, the screening conditions were set to QED > 0.34.

#### 2.5.2 Identification of components and EMs-Associated targets

The Swiss Target Prediction (Daina et al., 2019) (http://swisstargetprediction.ch) and Similarity Ensemble Approach (SEA, https://sea.bkslab.org) online platforms were used to forecast the component-related targets (Keiser et al., 2007). By employing “Endometriosis” as the search term, the top 25% of outcomes from the GeneCards database (Stelzer et al., 2016) (https://www.genecards.org) and all outcomes from the Online Mendelian Inheritance (OMIM) database (McKusick, 2007) (https://www.omim.org) were chosen. After merging and deduplicating the results, they were used as the EMs-related targets. Finally, Origin8.0 software was used to identify the intersection of component-associated and EMs-associated targets, and a Venn diagram was constructed.

#### 2.5.3 Construction of the component–target–disease and protein–protein interaction (PPI) networks

The active components, intersection targets, and disease data were imported into Cytoscape 3.5.1 software (Doncheva et al., 2018) to build the “component–target–disease” interaction network. The top 10 core components were identified based on the degree of each node. The STRING database (Szklarczyk et al., 2020) (https://cn.string-db.org) was utilized to retrieve the intersection targets and obtain the interaction relationship data between the targets. These data were then entered into Cytoscape, with the confidence level set at 0.4, to draw the PPI network and rank them according to their degree value.

#### 2.5.4 Gene ontology (GO) functional enrichment and Kyoto Encyclopedia of genes and genomes (KEGG) pathway enrichment analyses

KEGG enrichment analysis of core targets was performed using R Studio software (Li et al., 2021), and GO analysis was obtained using the WeChat online platform (http://www.bioinformatics.com.cn). According to *p* values, the top 10 biological processes, cellular components, molecular functions, and the top 30 KEGG signaling pathways were selected for visualization in bar charts or bubble charts.

#### 2.5.5 Molecular docking

The RCSB Protein Data Bank (Zardecki et al., 2021) (RCSB PDB, https://www1.rcsb.org) was used to acquire crystal structures of target proteins, and several filters were applied (Liu et al., 2022a), including 1) restriction of species origin to “Homosapiens,” 2) selection of high-resolution structures (resolution ≤2.5 Å), 3) preference for protein structures obtained through x-ray single-crystal diffraction scanning, and 4) consideration of the protein’s structure (single-stranded, double-stranded, or other) based on the number of polymers present in the organism. Proligands, crystallization water, and irrelevant protein chains in the protein structure were removed using PyMOL 2.3.0 software (Rosignoli and Paiardini, 2022). The structures of the core components were retrieved from the PubChem database and imported into the Chem3D software (version 2020) to minimize and optimize energy. Subsequently, AutoDockTools 1.5.6 software (Liu Y. et al., 2022) was used to add hydrogen atoms to the optimized proteins and ligands, and the torsion bonds of the small-molecule ligands were also selected. The docking area was predicted according to the original active pocket of the protein using AutoDock Tools. AutoDock Vina 1.2.0 software (Eberhardt et al., 2021) was then used for docking and calculating the binding energy; the lamarckian genetic algorithm and semi-flexible docking were chosen, default exhaustiveness was set to 8, and maximum output was set to 9. Finally, Discovery Studio 2020 and Pymol software were used to analyze and visualize the molecular docking results.

#### 2.5.6 Molecular dynamics simulation

The Gromacs 2022.3 software (Van Der Spoel et al., 2005; Abraham et al., 2015; Eberhardt et al., 2021) was employed to conduct molecular dynamic simulations. Small molecules were preprocessed using AmberTools22 to incorporate a general AMBER force field. Gaussian 16 W was applied to hydrogenate small molecules and estimate the restrained electrostatic potential (Sasitha and John, 2021). The obtained potential data were integrated into the topology file of the molecular dynamics system. The procedures were performed under static conditions, the temperature was set to 300 K, and the atmospheric pressure was set to 1 bar. The Amber99sb-ildn with water molecules (Tip3p water model) was selected as the solvent. The simulated system achieved charge neutrality by introducing suitable quantities of Na+. The system employed the steepest descent method to minimize energy, followed by the 100,000 steps of isothermal isovolumic ensemble and isothermal isobaric ensemble equilibria. The coupling constant was set at 0.1 ps, and the duration was 100 ps. A free molecular dynamics simulation was conducted, encompassing 5000,000 steps, the step length was set to 2 fs, and the total duration was 100 ns. Subsequently, the trajectory was analyzed, enabling the calculation of various parameters, such as root-mean-square variance (RMSD), and protein rotation radius of each amino acid trajectory, combined with free energy, free-energy topography, and other relevant data.

### 2.6 Cell experiments

#### 2.6.1 Cell culture

Eutopic endometrial tissue and cyst walls of ovarian endometriotic cysts were collected from patients with EMs who received laparoscopy combined with hysteroscopy at our hospital. This protocol was approved by the Ethics Committee of The First Affiliated Hospital of Guangzhou University of Chinese Medicine (NO. K [2021] 053). Primary human endometrial stromal cells (ESCs) were obtained using an improved method (Zhou et al., 2019). The tissue was cleaned and then cut into fragments smaller than 1 cm^3^. The endometrial tissue was treated with an approximately equal volume of type I collagenase (1 g/mL) and digested at 37°C for 30 min. The capsule wall tissue was digested with approximately twice the volume of type I collagenase (lg/mL) for 60 min. The digestion was then terminated with twice the volume of Dulbecco’s Modified Eagle Medium (DMEM)–F12 containing 10% FBS. The digestive fluid was first filtered through a 100-mesh sieve and then through a 400-mesh sieve. ESCs were contained in the filtrate. The filtrate was centrifuged at 1,000 rpm for 10 min, and the supernatant was discarded. The remaining supernatant was resuspended and precipitated using a complete medium. ESCs were seeded in a 6 cm diameter culture dish at a density of 1–2 × 10^5^ cells/mL, and 3 mL of complete medium was added to each dish. The cells were then cultured in a cell incubator at 5% CO_2_ and 37°C. The medium was changed 48 h after the primary cells were extracted and then once every 3–5 days, depending on the growth of the cells. The cells were subcultured upon reaching a cell growth fusion of 80%–90%. Third-to fifth-generation ESCs were used for this experiment.

#### 2.6.2 CCK-8 assay

Ectopic endometrial stromal cells (ecESCs) that were in the logarithmic growth phase and exhibited robust growth were seeded in 96-well plates at a density of 3 × 10^3^ cells/well. Subsequently, the cells were incubated overnight at 37°C in a 5% CO_2_ incubator. The cells were then divided into five groups: the ecESCs (10% blank serum), low LSNYP dose (10% LSNYP-containing serum: blank serum = 1:4), middle LSNYP dose (10% LSNYP-containing serum: blank serum = 3:2), high LSNYP dose (10% LSNYP-containing serum: blank serum = 1:0), and danazol (10% danazol-containing serum: blank serum = 1:0) groups. After the corresponding serum was added, the cells were cultured for 24, 48, and 72 h, and 10 μL of CCK8 was added to each well and incubated at 37°C for 1 h. The absorbance of each well was quantified using a microplate reader set to measure the optical density (OD) at a wavelength of 450 nm. Cell proliferation rate = (OD value of experimental group - OD value of blank well)/(OD value of model group - OD value of blank well) × 100%. CCK-8 assay results showed that 72 h was the optimal time for the drug-containing serum to inhibit ecESC proliferation.

#### 2.6.3 Cell adhesion assay

Cells were seeded in 6-well plates at a density of 5 × 10^5^ cells/well and categorized as 1.6.2. Following a 72-h intervention with drug-containing serum, the cells were harvested using trypsin digestion, and the concentration was diluted to 6 × 10^4^ cells/mL using the DMEM/F12 medium. Subsequently, 50 μL of Matrigel with a final concentration of 1 mg/mL was vertically added to the center of a 96-well plate and incubated at 37°C for 4–5 h until it solidified into a gelatinous state. The remaining medium was removed, and 100 μL of each group’s cell suspension was added. The cells were subsequently cultured in an incubator at 37°C and 5% CO_2_ for 1 h. Finally, the non-adherent cells were washed off with DMEM/F12 medium, 10 μL of CCK-8 was added to each well and cultured at 37°C for 3 h, and the OD was calculated.

#### 2.6.4 Transwell invasion assay

The cells were administered drug-containing serum for 72 h, and the cell concentration was adjusted to 3 × 10^5^/mL following digestion. Subsequently, 800 μL of DMEM/F12 medium containing 10% FBS was introduced into a 24-well plate and positioned within the transwell chamber. A 100 μL volume of Matrigel, with a final concentration of 0.5 mg/mL, was added vertically at the bottom of the upper chamber. Once the Matrigel had solidified into a gel, a 200 μL suspension of cells from each group was introduced into the upper chamber of the transwell and incubated at 37°C and 5% CO_2_ environment. The transwell plate was removed, and the chamber was washed with phosphate-buffered saline (PBS). The cells were fixed with 70% ice-cold ethanol solution for 1 h, stained with 0.5% crystal violet staining solution, and incubated at 25°C for 20 min. After rinsing with PBS, the non-migrating cells in the upper chamber were gently wiped off using cotton balls and then observed and captured under a microscope.

#### 2.6.5 Cell scratch test

Using a marker pen, the back surface of a 6-hole plate was marked with evenly spaced lines at intervals ranging from 0.5–1 cm, ensuring that each hole was intersected by at least three lines (Wang and Li, 2021). The digested cells were seeded in 6-well plates at a density of 1 × 10^6^ cells/well. Once the cell density exceeded 90%, a horizontal scratch was created perpendicular to the back of the plate. Next, the cells were washed thrice with PBS and treated with a serum-free medium, and images were captured at 0 h. Subsequently, they were cultured at 37°C in a 5% CO_2_ incubator and photographed at 24 h.

#### 2.6.6 Lentiviral transfection

Cells were inoculated into 6-well plates at a density of 5 × 10^4^ cells/well. When the cell attachment rate was approximately 30%, the lentivirus rLV-shRNA containing HIF1A was introduced to establish a cell line with low expression of HIF1A. Subsequently, the cells were administered with drug serum and divided into six groups: the euESCs (10% blank serum), ecESCs (10% blank serum), high LSNYP dose (10% LSNYP-containing serum: blank serum = 1 : 0), si-NC (10% LSNYP-containing serum + si-NC), si-HIF1A (10% LSNYP-containing serum + si-HIF1A), and danazol (10% danazol-containing serum: blank serum = 1 : 0) groups. After the cells were cultured in the drug-containing serum for 72 h, the cell culture supernatant and cells were collected for experimentation.

#### 2.6.7 Western blotting

Cells in each group were added with radioimmunoprecipitation assay lysate, placed on ice for 10 min, and centrifuged at 12,000 rpm for 5 min at 4°C. Protein concentration was determined using the bicinchoninic acid protein-concentration determination kit. Approximately 40 μg of protein was boiled in a boiling water bath for 10 min and then added to the sample hole with MAKER. The proteins were separated by sodium dodecyl sulphate-polyacrylamide gel electrophoresis, and electrophoresis was conducted under a constant pressure of 80 V until the bromophenol blue indicator reached the junction of the concentrated and separation gel, after which the constant pressure was changed to 120 V until the bromophenol blue reached the bottom of the gel. This process took approximately 1.5 h. The isolated proteins were transferred onto a polyvinylidene fluoride membrane and blocked with 5% skim milk powder at 25°C for 2 h. The primary antibodies HIF-1A, EZH2, ANTXR2, YAP1, CD44, β-Catenin, and GAPDH, were diluted with Tris Buffered Saline with Tween 20 (TBST) at ratios of 1:1,000, 1:2,000, 1:1,000, 1:10,000, 1:3,000, 1:10,000, and 1:1,000, respectively. These antibodies were then added to the sample and allowed to react overnight at 4°C. The next day, these membranes were washed five times with TBST, and an HRP-labeled secondary antibody (1:10,000) was added. After another round of washing, the enhanced chemiluminescence reagent was applied for development. A gel imaging system was used for scanning exposure and filming. Finally, we used the Image-Pro Plus (IPP) software to calculate the gray value of the film (Mei et al., 2016).

### 2.7 Animal experiments

#### 2.7.1 Experimental model of EMs and treatment

Twenty-four rats were randomly selected to establish the EMs model by autotransplantation in accordance with a previously described method (Oner et al., 2010). To synchronize their estrous cycles, the rats were intramuscularly injected with E2 benzoate (0.05 mg/kg/day) for 5 days prior to the operation. Subsequently, the rats were anesthetized and a 2 cm incision was made in the middle of the lower abdomen to expose the uterus. The left uterus was excised after ligation using 1# silk threads, and the surrounding adipose tissues were removed. The isolated uterus was cut open longitudinally and divided into two 5 mm × 5 mm pieces. Afterward, the samples were stitched to the right and left abdominal walls near the great vessels with 0/5 absorbable sutures. The endometrial surface of each implant faced the abdominal wall. After the surgery, the rats were treated with penicillin sodium by injection (160,000 units/kg/d) to prevent infection and E2 benzoate to promote the growth of the ectopic endometrium for 5 days. In addition, six rats were included in the sham-operated group, their abdomens were only opened and stitched, but their uteruses were not transplanted.

On the 29th postoperative day, the rats were divided into five groups: sham, model, low dose of LSNYP (L-LSNYP), high dose of LSNYP (H-LSNYP), and danazol. The L-LSNYP group were orally administered a double dose of LSNYP, and the H-LSNYP and danazol groups were subjected to the same procedure, as described in 2.3. The sham and model groups were administered an equal volume of normal saline. All rats were administered doses once per day for four consecutive weeks. Finally, the rats were euthanized, and their uterine tissues and endometriotic tissues were resected for pathological observations and other analyses.

#### 2.7.2 Hematoxylin and eosin (H&E) staining

H&E staining was used for observing histopathology in endometriotic tissues. Tissues were fixed in a 4% paraformaldehyde solution and dehydrated with ethanol, then cleared with xylene and embedded in paraffin. Subsequently, the sections were cut from the paraffin blocks, then routinely dewaxed and hydrated. The hematoxylin was applied as a background staining. Hydrochloric acid and ethanol solution were used for color separation before counterstaining with eosin. Finally, the sections were rinsed, dehydrated, cleared, and mounted for microscopic observation.

#### 2.7.3 Quantitative real-time PCR (qPCR)

Total RNA from the tissues was extracted using the Trizol reagent. The HiScript^®^ II Q Select RT SuperMix was used to reverse transcribe the RNA into complementary DNA. The qPCR was performed with the SYBR Green Master Mix. PCR products were detected by fluorescence quantitative PCR equipment (Applied Biosystems, California, United States). The PCR program was performed as follows: pre-denaturation at 95°C for 10 min, denaturation at 95°C for 15 s, annealing at 60°C for 60 s and extension at 95°C for 15 s, for 40 cycles. The melting curve analysis of the amplification products was performed at 95°C for 15 s, at 60°C for 60 s, and at 95°C for 15 s again. *Gapdh* was used as an internal reference. Relative mRNA expression levels were calculated using the 2^−ΔΔCt^ method. The primers (Table 2) were obtained from Sangon Biotech (Shanghai) Co., Ltd.

**TABLE 2:** Primer sequences.

Gene	Primer	Sequence (5’-3’)	Size
*Gapdh*	Forward	AGT​CTA​CTG​GCG​TCT​TCA​CC	225 bp
	Reverse	CCA​CGA​TGC​CAA​AGT​TGT​CA	
*HIf1a*	Forward	GCG​GCG​AGA​ACG​AGA​AGA​AAA​ATA​G	129 bp
	Reverse	GAA​GTG​GCA​ACT​GAT​GAG​CAA​G	
*Ezh2*	Forward	GCA​CAC​TGC​AGA​AAG​ATC​CA	224 bp
	Reverse	AGG​TAG​CAC​GGA​CAC​TGC​TT	
*Antxr2*	Forward	AAC​GGG​ATT​GCA​GCC​ATC​ATA​GC	150 bp
	Reverse	CTC​TTC​CTC​CTC​CTC​CTC​CTT​TGG	
*Yap1*	Forward	AGC​CGC​CTG​AGC​CCA​AGT​C	105bp
	Reverse	GGG​AGG​CTG​GAG​ACG​AGT​GAG	
*Cd44*	Forward	AAA​CAC​CAC​CCA​AGA​GGC​AAG​AAG	110 bp
	Reverse	TGA​CTC​CGT​ACC​AGG​CAT​CTT​CG	
*Ctnnb1*	Forward	TAC​CGC​TGG​GAC​CCT​ACA​CAA​C	137 bp
	Reverse	GCG​TGG​TGA​TGG​CGT​AGA​ACA​G	

#### 2.7.4 Immunofluorescence analysis

After antigen retrieval was performed with 0.01 M sodium citrate buffer, the sections were treated with normal goat serum for 30 min to block nonspecific antibody binding and were incubated overnight at 4°C with primary antibodies against HIF1A (1:200), EZH2 (1:200), ANTXR2 (1:300), YAP1 (1:200), CD44 (1:200), and β-Catenin (1:300). On the next day, the sections were thoroughly washed with PBST three times for 3 min each, then the secondary antibody was added and incubated at 37°C for 1 h. Finally, the nuclei were counterstained with 4,6-diamino-2-phenyl indole (DAPI) for 5 min, and the slices were mounted with an anti-fluorescence quencher. Three fields were selected randomly from each section using a BX53 microscope (Olympus, Japan). The mean fluorescence intensity was analyzed with ImageJ software.

### 2.8 Statistical analysis

Data analysis was performed using SPSS version 27.0. Data are expressed as mean ± standard deviation (SD). One-way analysis of variance was used to compare multiple groups. *P* < 0.05 was considered statistically significant.

## 3 Results

### 3.1 Results of UPLC-Q-TOF/MS and drug-likeness screening

**FIGURE 1:**
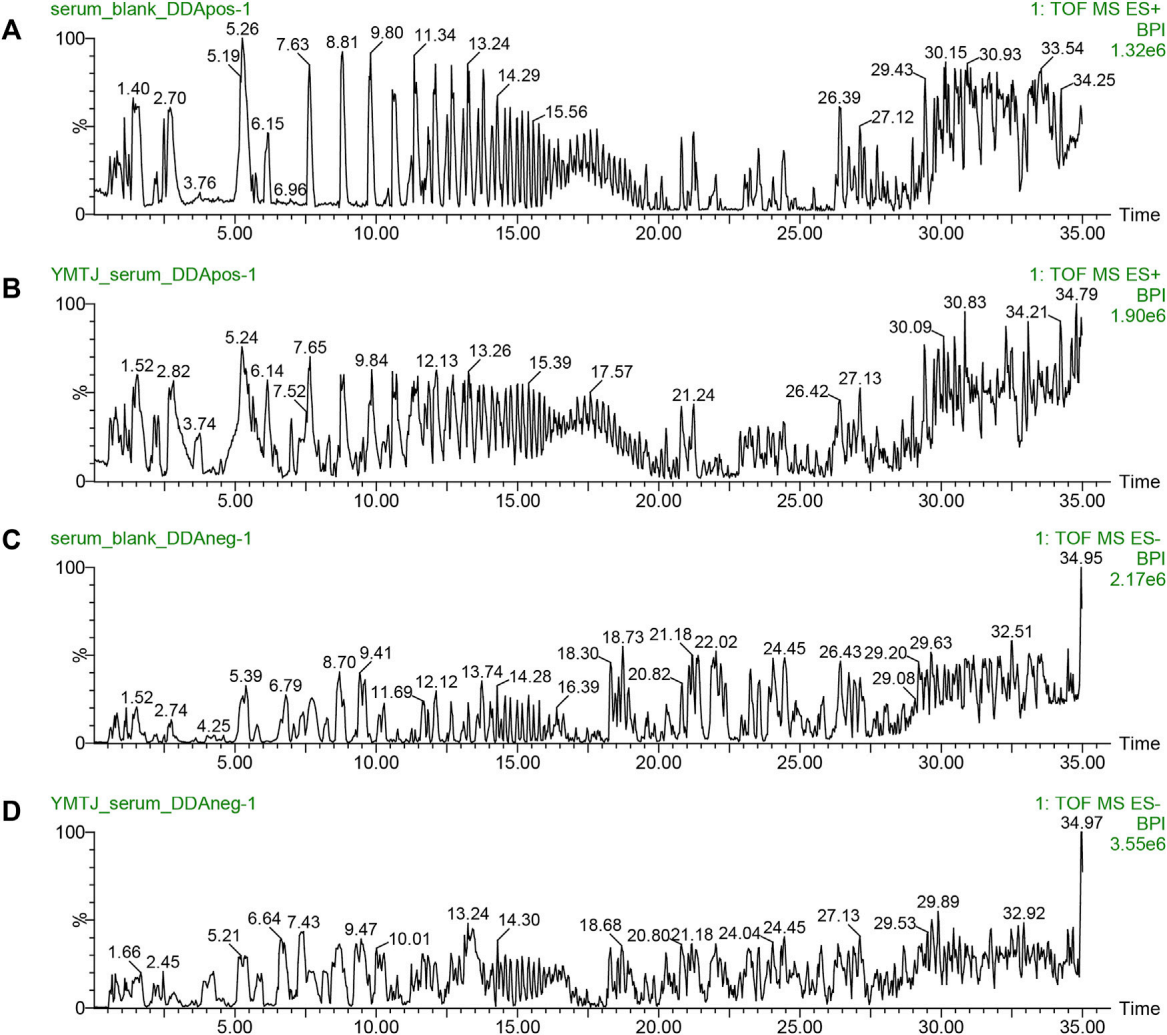
Ion flow diagrams of serum samples. The base peak intensity (BPI) chromatograms of in positive ion mode: **(A)** blank serum and **(B)** drug serum; BPI chromatograms in negative ion mode: **(C)** blank serum, **(D)** drug serum.

The ion flow diagrams of the serum samples in positive and negative ion modes are exhibited in Figure 1. Components present in drug-containing serum samples and LSNYP, but absent in blank serum, were identified as prototype components. In our previously unpublished *in vitro* experiment with LSNYP samples, 185 components were detected under the same mass spectrum conditions (Supplementary Table S1). Through a new comparison, 45 prototype components (Supplementary Table S2) were confirmed to be absorbed in the blood, based on the retention time, primary MS data, and secondary MS data. These compounds underwent further screening using the PubChem database and ADMET lab2.0, and 34 compounds (Table 3) were identified as active compounds in LSNYP for subsequent analysis.

**TABLE 3:** Potential active components of LSNYP.

Component name	QED	Component name	QED
Boldine	0.888	Dehydrocorydaline	0.661
Isoboldine	0.888	Vanillin	0.648
Stepholidine	0.888	p-Hydroxybenzoic acid	0.61
Rotundine	0.841	Macrophylloside A	0.598
Tetrahydropalmatine	0.841	1-(4-nonylphenoxy)-2-propanol	0.592
Lysergol	0.817	Cryptotanshinone	0.579
Norbracteoline	0.795	Isocryptotanshinone	0.579
Norisoboldine	0.795	Ethyl 4-methoxycinnamate	0.56
Senkyunolide F	0.78	Senkyunolide P	0.539
13-Methyl-dehydrocorydalmine	0.734	Hydroxylinderstrenolide	0.527
Moupinamide	0.716	Byzantionoside B	0.507
17β-Hydroxy--2-oxa-5α-androstan-3-one	0.698	Nikoenoside	0.485
Lindenenol	0.695	1,2-Dihexyloxybenzene	0.463
Shizukanolide A	0.694	Protocatechualdehyde	0.448
Deoxyschizandrin	0.676	Neocryptotanshinone	0.447
Palmatine	0.675	Tanshinoldehyde	0.396
n-Butylidenephthalide	0.667	Dehydrocostuslactone	0.363

### 3.2 Network pharmacological target prediction

**FIGURE 2:**
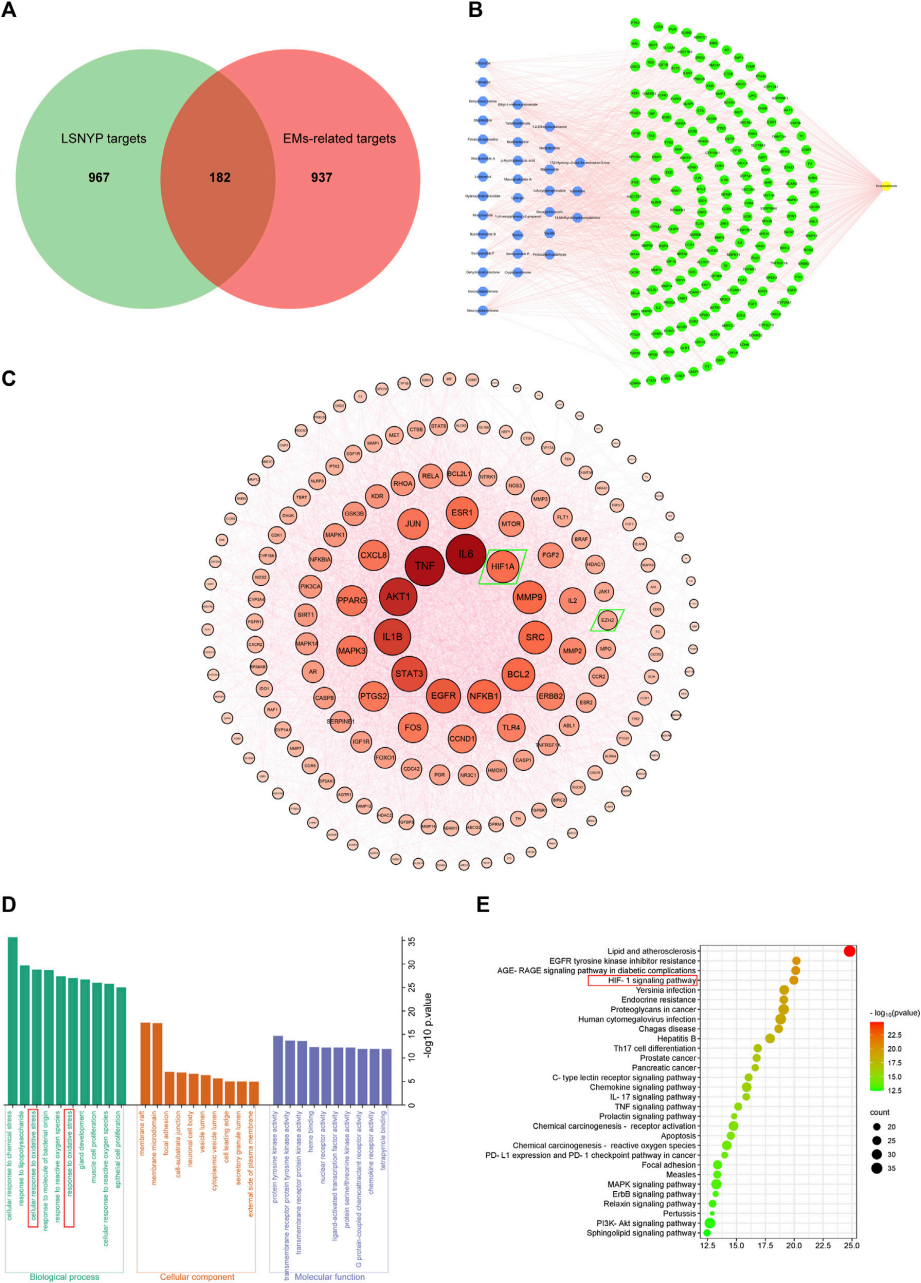
Network pharmacological target prediction. **(A)** Venn diagram of LSNYP targets and EMs-related targets. **(B)** Component–target–disease network. **(C)** PPI network of key targets. **(D)** GO enrichment analysis. **(E)** KEGG enrichment analysis.

Based on SwissTargetPrediction and SEA databases, 1149 (Supplementary Table S3) potential targets were acquired after merging and eliminating duplicates. By incorporating the GeneCards (top 25%) and OMIM databases, 1119 (Supplementary Table S4) targets associated with EMs were obtained. A Venn diagram (Figure 2A) was constructed, and 182 (Supplementary Table S5) key targets of LSNYP in EMs treatment were identified. The component–target–disease network (Figure 2B) revealed that, on average, each compound was associated with 20 targets. Table 4 presents information on the top 10 compounds based on their degree value in the intersection network. A higher degree indicates a potentially greater number of associated targets (Liu et al., 2023a; Jiao et al., 2023). Notably, ethyl 4-methoxycinnamate, moupinamide, boldine, 1,2-dihexyloxybenzene, and neocryptotanshinone exhibited degrees ≥33, indicating that they may be of great significance in the treatment of EMs. The PPI diagram (Figure 2C) showed that the core proteins included IL-6, EGFR, HIF1A, MMP9, and EZH2, which exhibit significant associations with hypoxia, adhesion, invasion, and angiogenesis. 2,425 biological process terms, such as cellular response to chemical stress, oxidative stress, and epithelial cell proliferation; 75 cellular component terms, including membrane raft, focal adhesion, and cell-substrate junction; and 154 molecular function categories focused on nuclear receptor and transcription factor activity, were identified for GO analysis. The top 10 terms were visually represented (Figure 2D). Additionally, KEGG analysis was performed, the top 30 pathways related to EMs were visualized (Figure 2E), including oxidative stress-related pathways, such as the HIF-1 signaling pathway, AGE–RAGE signaling pathway in diabetic complications; inflammation-related pathways, such as the TNF signaling and IL-17 signaling pathways; and cell proliferation-related pathways, such as the pI3K-Akt signaling and MAPK signaling pathways.

**TABLE 4:** Topological parameters of active component nodes (top10).

Component name	Degree	Betweenness centrality	Closeness centrality
Ethyl 4-methoxycinnamate	46	0.026342569	0.411992263
Moupinamide	46	0.026411848	0.413592233
Boldine	34	0.012370107	0.393715342
1,2-Dihexyloxybenzene	33	0.014842993	0.392265193
Neocryptotanshinone	33	0.011874793	0.392265193
1-(4-nonyl phenoxy)-2-propanol	32	0.013579075	0.387978142
Deoxyschizandrin	31	0.010996563	0.387978142
Isoboldine	30	0.009408465	0.389396709
Senkyunolide F	29	0.012241345	0.386569873
Palmatine	27	0.007814418	0.378330373

The etiology of EMs remains incompletely understood; however, the widely-accepted theory of retrograde menstruation posits that shed-off endometrial tissues first lose blood supply and undergo hypoxic stress in the abdominal cavity. Within this microenvironment, upregulation of HIF1A serves as a key driver of endometriosis, activating multiple signaling pathways (Wu et al., 2019). The HIF1A/EZH2/ANTXR2 pathway involves deep epigenetic changes. HIF1A suppresses EZH2 expression and reduces H3K27 trimethylation, leading to abnormal ANTXR2 expression in EMs, and activating proteins like YAP1, β-Catenin, CD44, COL5A2 and VEGFC (Lin et al., 2019). Additionally, YAP1 and β-Catenin, serving as crucial regulatory factors, increase downstream targets such as StAR, COX-2, IL-1β, VEGF, MMP-9, and BCL2 (Liu et al., 2016; Song et al., 2016; Zhang et al., 2016; Lin et al., 2017), directly or indirectly promoting E2 and PGE synthesis and activating various pathways like EGFR, NF-kB, and TNFα pathways in EMs (Banu et al., 2009; Lin et al., 2017). These help in the development of the complex gene regulatory networks and facilitate the development of EMs. Through GO and KEGG analysis, our study identified the response to oxidative stress and the HIF-1 signaling pathway. HIF1A, EZH2, and subsequent direct or indirect downstream targets like IL-1β, COX-2, StAR, MMP-9, TNF, and BCL2 were identified as core targets. Because several key regulators and so many important downstream targets are involved in this pathway, we hypothesized that LSNYP may treat EMs by improving the hypoxic environment. Therefore, we chose the HIF1A/EZH2/ANTXR2 pathway for experimental verifications and molecular docking.

### 3.3 Cell experiments

#### 3.3.1 Effect of LSNYP on the proliferation and migration of ecESCs

**FIGURE 3:**
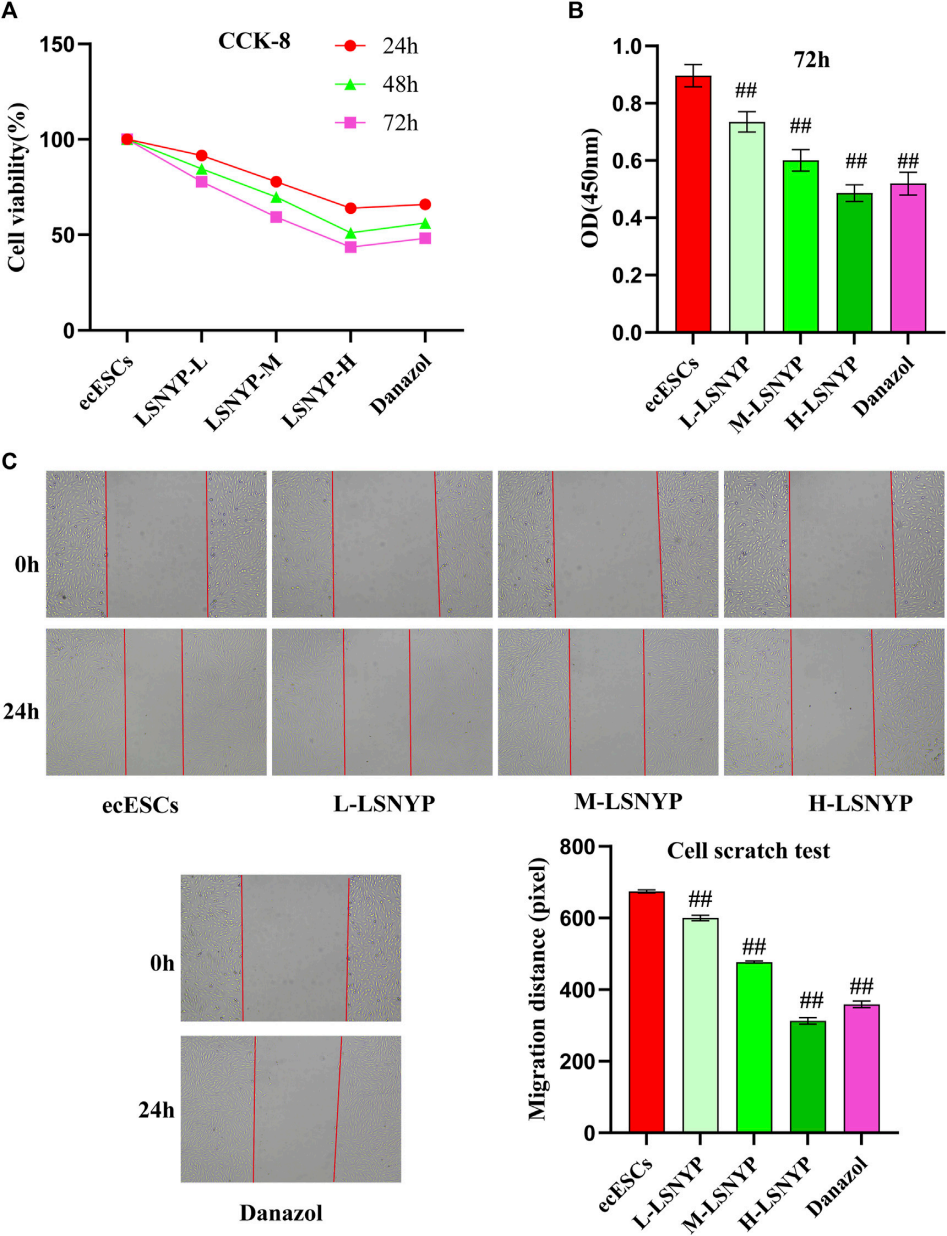
Effect of LSNYP on the proliferation and migration of ecESCs. **(A)** Effect of different intervention times on cell viability. **(B)** The impact of LSNYP on the viability of ecESCs 72 h after treatment. **(C)** Cell scratch experimental results. Compared with the ecESCs group, ^##^ *P* < 0.01.

The inhibition rate of LSNYP on cellular activity increased with a prolonged intervention period (Figure 3A), and serum with varying drug concentrations had the potential to diminish cell viability after 24, 48, and 72 h of intervention. Nevertheless, the L-LSNYP, M-LSNYP, and H-LSNYP significantly differed only at 72 h when compared with the ecESCs group (*P* < 0.01; Figure 3B). The migration distance of cells was assessed after 72 h of serum intervention, and the results showed that the migration distance of cells in the LSNYP and danazol groups was significantly lower than that in the ecESCs group (*P* < 0.01; Figure 3C).

#### 3.3.2 Effect of LSNYP on the adhesion and invasion of ecESCs

**FIGURE 4:**
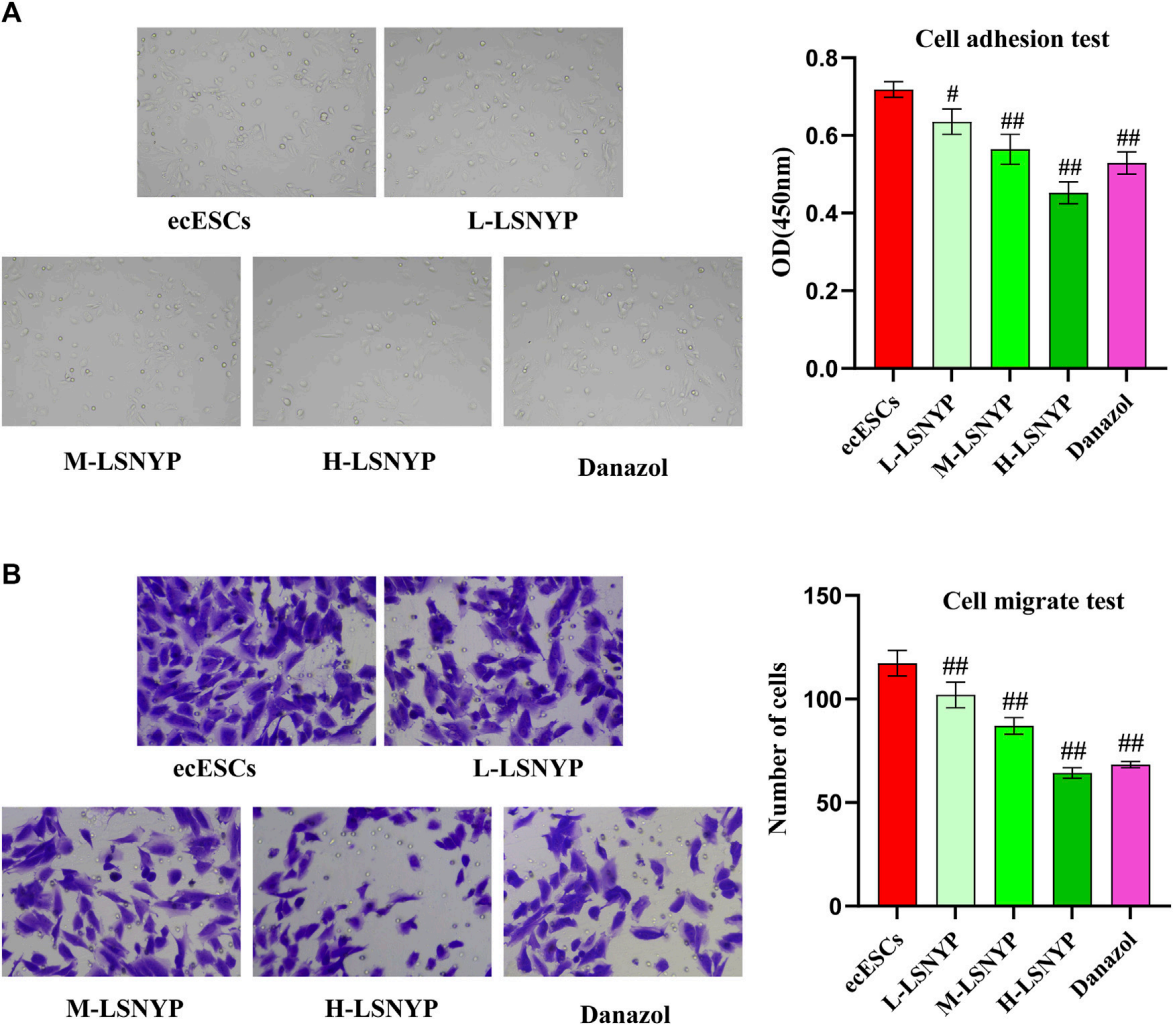
Effects of LSNYP on adhesion **(A)** and invasion **(B)** of ecESCs. Compared with the ecESCs group, ^#^ *P* < 0.05, ^##^ *P* < 0.01.

After 72 h of LSNYP treatment, ecESCs adhesion was observed. The results showed that the cell migration capability in the H-LSNYP and danazol groups was significantly diminished compared to that in the ecESCs group (Figure 4A) (*P* < 0.01). Additionally, invasive cells were detected, and the number of invasive cells in the LSNYP and danazol groups was significantly lower than that in the ecESCs group (*P* < 0.01; Figure 4B).

#### 3.3.3 Effect of LSNYP on HIF1A/EZH2/ANTXR2 pathway in ecESCs

**FIGURE 5:**
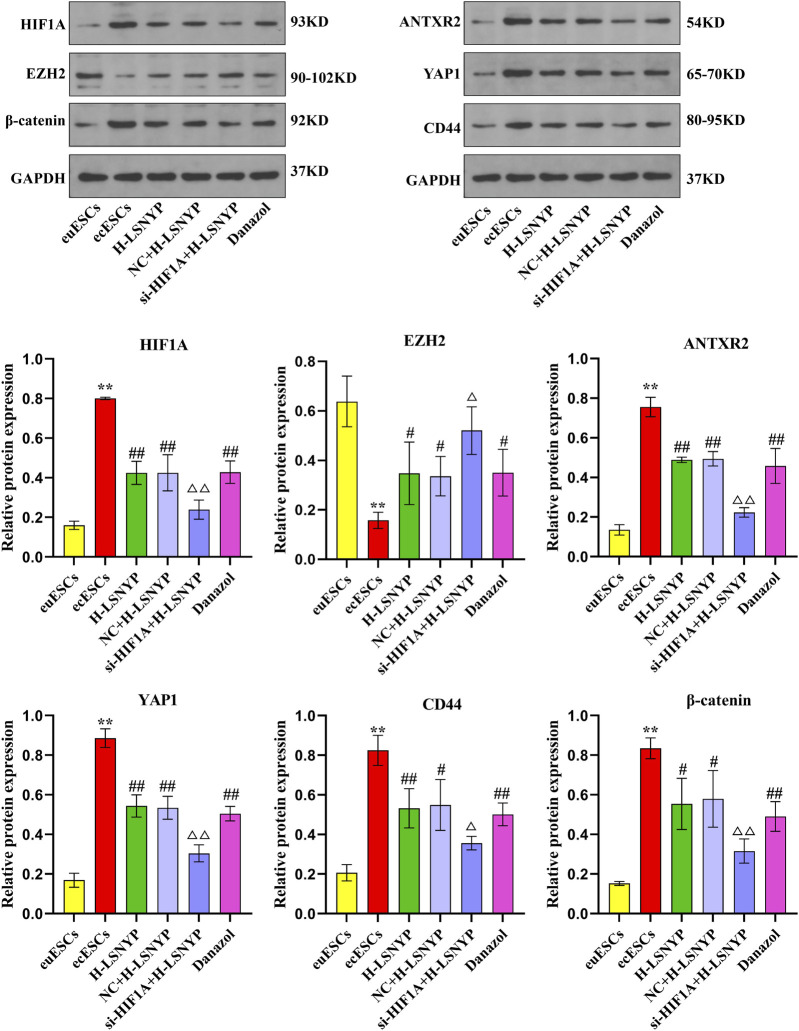
Effect of LSNYP on the HIF1A/EZH2/ANTXR2 pathway determined using western blotting. Compared with the euESCs group, ^**^ *P* < 0.01; compared with the ecESCs group, ^#^ *P* < 0.05, ^##^ *P* < 0.01; compared with the NC + H-LSNYP group, ^△^ *P* < 0.05, ^△△^ *P* < 0.01, *n* = 3.

Based on the aforementioned series of experiments, H-LSNYP exhibited a superior inhibitory effect on cells following a 72-h intervention. Consequently, H-LSNYP were chosen as the optimal intervention condition for western blotting (Figure 5). Compared to the euESCs group, the ecESCs group demonstrated a significant increase in the expression levels of HIF1A, ANTXR2, YAP1, CD44, and β-catenin (*P* < 0.01), whereas the expression level of EZH2 was significantly reduced (*P* < 0.01). Following treatment with the H-LSNYP and danazol, the expression levels of HIF1A, ANTXR2, YAP1, CD44, and β-catenin were decreased (*P* < 0.05 or *P* < 0.01), whereas the expression level of EZH2 was increased (*P* < 0.05). Compared to the NC + H-LSNYP group, the si-HIF1A + H-LSNYP group showed lower expression levels of HIF1A, ANTXR2, YAP1, CD44, and β-catenin (*P* < 0.05 or *P* < 0.01), with a higher expression level of EZH2 (*P* < 0.05).

### 3.4 Animal experimentations

#### 3.4.1 Histopathological analysis

**FIGURE 6:**
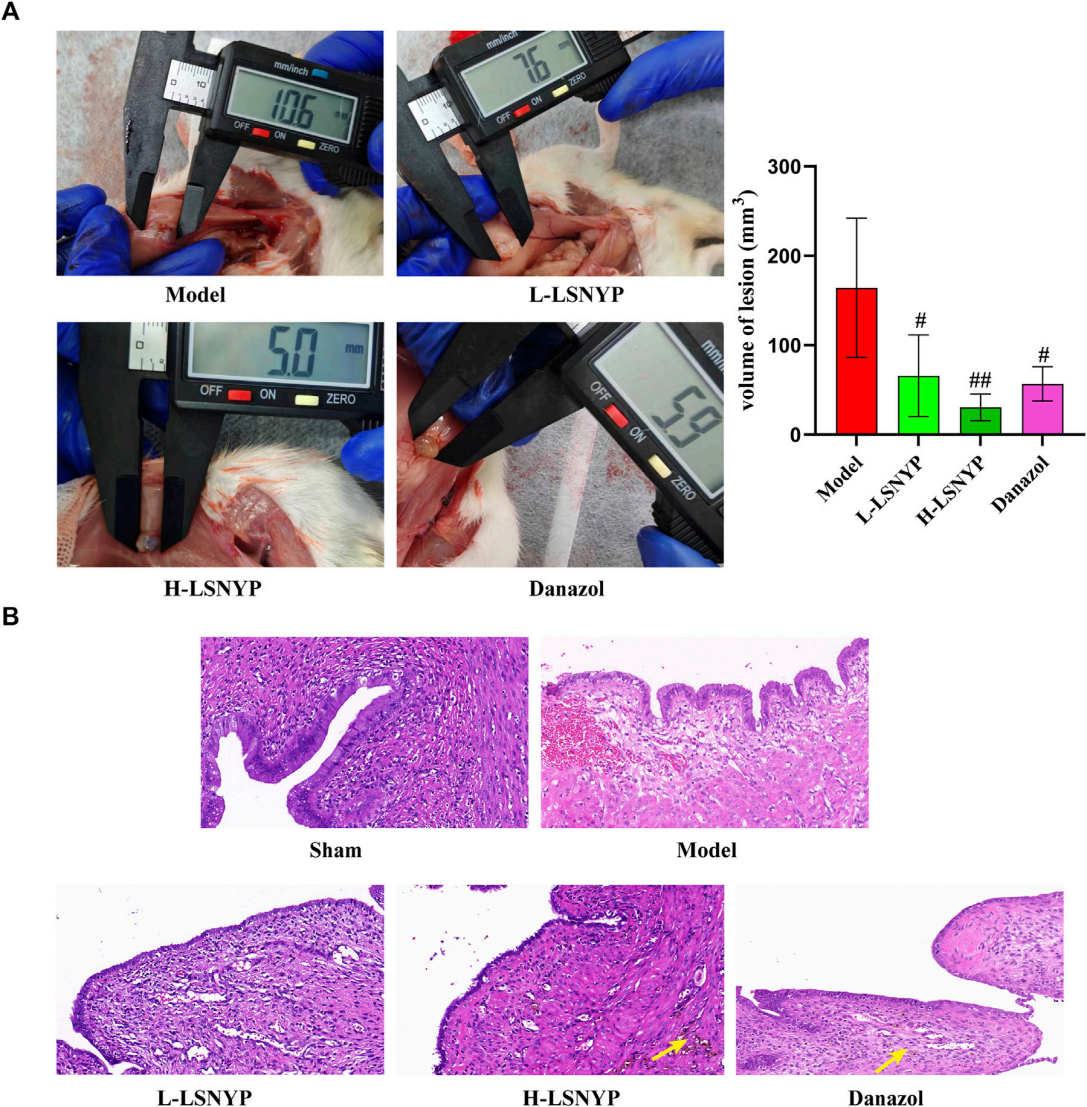
Effect of LSNYF on volume of lesion **(A)** and histopathology **(B)** in endometriotic tissues (×400). Compared with the model group, ^#^ *P* < 0.05, ^##^ *P* < 0.01, *n* = 6.

Macroscopic observation of endometriotic foci showed that there were cystic lesions containing clear fluid with vascularization visible on the surface and periphery. Partial lesions adhered to the intestines, but lysis was relatively easy. Than in the model group, smaller lesion volumes (Figure 6A) were observed in the LSNYP groups (*P* < 0.05 or *P* < 0.01). Endometriotic tissues (Figure 6B) in the sham group showed a monolayer of column-like epithelium with unaltered structures, even-sized, well-organized stromal cells, numerous glands, and few inflammatory cells. In comparison, the pathological results in the model group showed that epithelial cells were irregularly arranged, and some of them were structurally incomplete. There were increased, enlarged stromal cells and many inflammatory cells. The Danazol group exhibited thinner, more well-organized epithelial cells and fewer stromal cells and inflammatory cells after treatment. In addition, there were macrophages containing hemosiderin (represented by yellow arrows), which indicates that the phagocytic ability was good. The L-LSNYP group also showed a thinner columnar epithelium than the model and sham groups, but the improvements in inflammation and the increased number of macrophages were less than those in the Danazol group and the H-LSNYP group. The pharmacological effects in the H-LSNYP group were comparable to those in the Danazol group.

#### 3.4.2 Effect of LSNYP on the expression of mRNA related to HIF1A/EZH2/ANTXR2 pathway in endometriotic tissues

**FIGURE 7:**
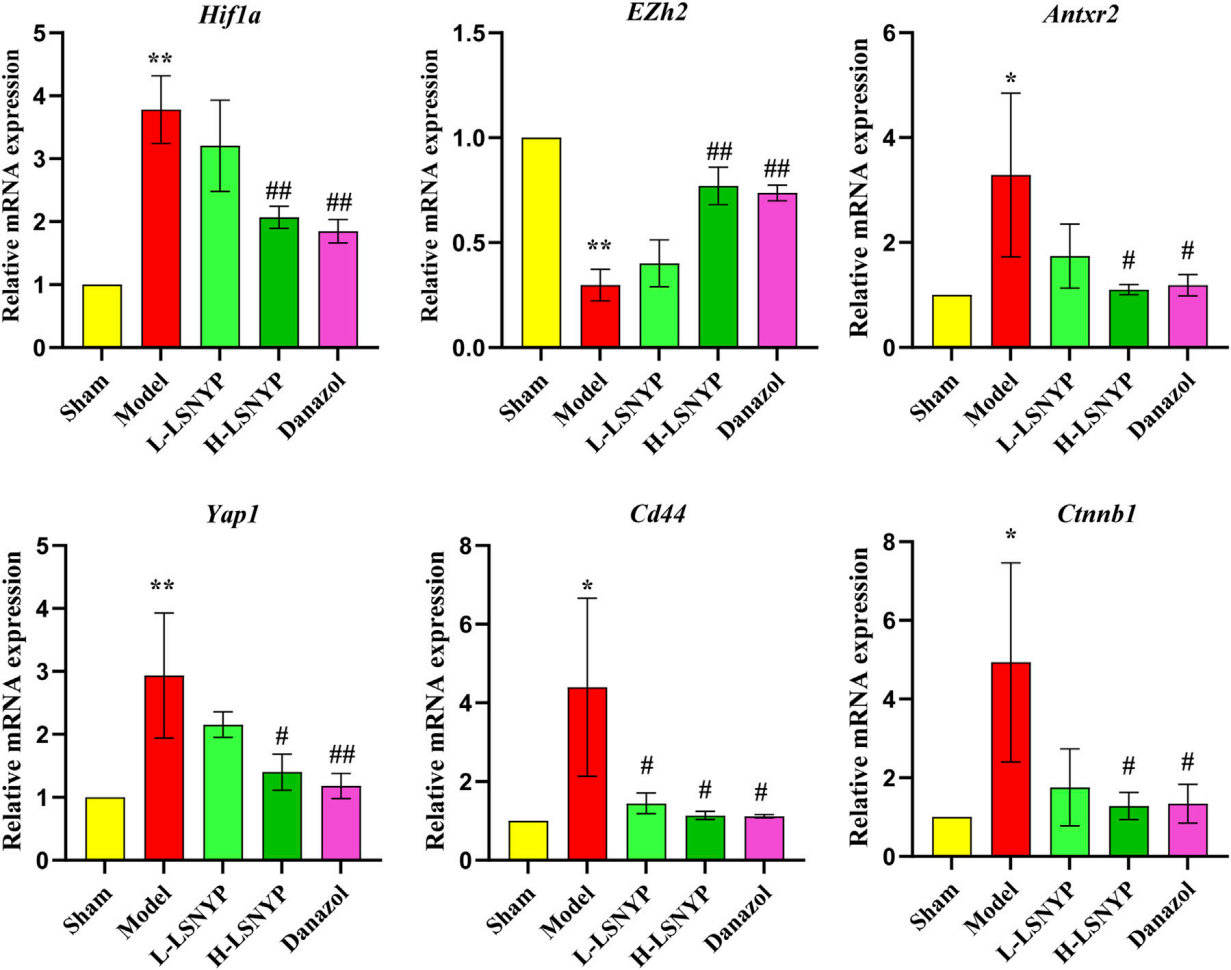
Effect of LSNYP on the expression of mRNA related to HIF1A/EZH2/ANTXR2 pathway in endometriotic tissues. Compared with the sham group, ^*^ *P* < 0.05, ^**^ *P* < 0.01; compared with the model group, ^#^ *P* < 0.05, ^##^ *P* < 0.01, *n* = 3.

The results of qPCR (Figure 7) showed that the expression of *Hif1a*, *Antxr2*, *Yap1*, *Cd44* and *Ctnnb1* mRNA increased significantly in the model group (*P* < 0.05 or *P* < 0.01), whereas *Ezh2* mRNA decreased significantly (*P* < 0.01). Compared with the model group, the expression of *Hif1a*, *Antxr2*, *Yap1*, *Cd44* and *Ctnnb1* mRNA decreased in the H-LSNYP group (*P* < 0.05 or *P* < 0.01), whereas the expression of *Ezh2* increased significantly after H-LSNYP intervention (*P* < 0.05).

#### 3.4.3 Effect of LSNYP on the expression of proteins related to HIF1A/EZH2/ANTXR2 pathway in endometriotic tissues

**FIGURE 8:**
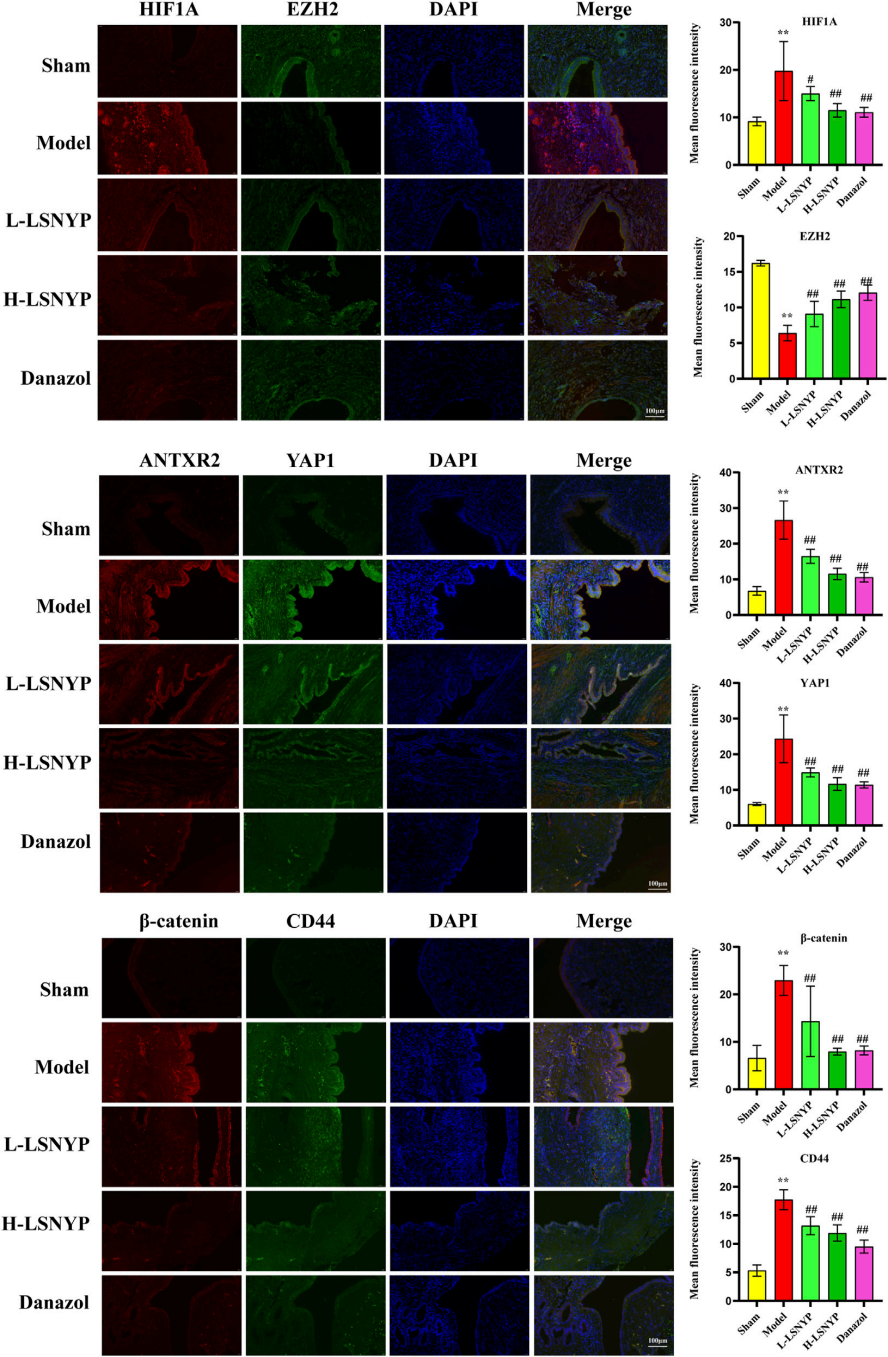
Effect of LSNYP on the HIF1A/EZH2/ANTXR2 pathway in endometriotic tissues were measured by immunofluorescence (400×). Compared with the sham group, ^**^ *P* < 0.01; compared with the model group, ^#^ *P* < 0.05, ^##^ *P* < 0.01.

The immunofluorescent staining results (Figure 8) showed that the model group demonstrated a significant increase in the expression levels of HIF1A, ANTXR2, YAP1, CD44, and β-catenin (*P* < 0.01) compared with the sham group, whereas the expression level of EZH2 was significantly reduced (*P* < 0.01). Following treatment with LSNYP and danazol, the expression levels of HIF1A, ANTXR2, YAP1, CD44, and β-catenin were decreased (*P* < 0.05 or *P* < 0.01), whereas the expression level of EZH2 was increased (*P* < 0.01).

### 3.5 Molecular docking and molecular dynamics simulation results

To further validate the relationship between LSNYP and the HIF1A/EZH2/ANTXR2 pathway, we conducted a molecular docking analysis between 34 components and the key proteins of the pathway. Information on the targets is listed in Table 5, and the docking binding energies are shown in Supplementary Table S6. Generally, small molecules can bind to proteins when the binding energy is less than −1.2 kcal/mol or −5 kj/mol. Notably, all the binding energies were lower than −3.6 kcal/mol, suggesting that LSNYP has a high possibility of inhibiting the HIF1A/EZH2/ANTXR2 pathway in EMs.

**TABLE 5:** The information of targets for molecular docking.

Target	PDB ID	Resolution	Structure	(X × Y × Z)/nm3	Center (X, Y, Z)
HIF1A	1H2K	2.15 Å	single stranded	33.3 × 50.7 × 29.7	34.2, 19.9, 30.7
EZH2	4MI0	2.00 Å	single stranded	18.0 × 23.0 × 24.0	24.0, 27.5, 10.0
ANTXR2	1SHT	1.81 Å	single stranded	17.6 × 17.2 × 15.0	49.2, 66.4, −0.5
YAP1	4REX	1.60 Å	single stranded	20.0 × 22.0 × 30.0	7.0, 48.0, 55.0
CD44	1UUH	2.20 Å	single stranded	19.0 × 18.0 × 18.0	−4.5, 13.0, 19.0
β-Catenin	1JDH	1.90 Å	single stranded	21.0 × 18.0 × 18.0	12.5, 7.0, 15.0

**FIGURE 9:**
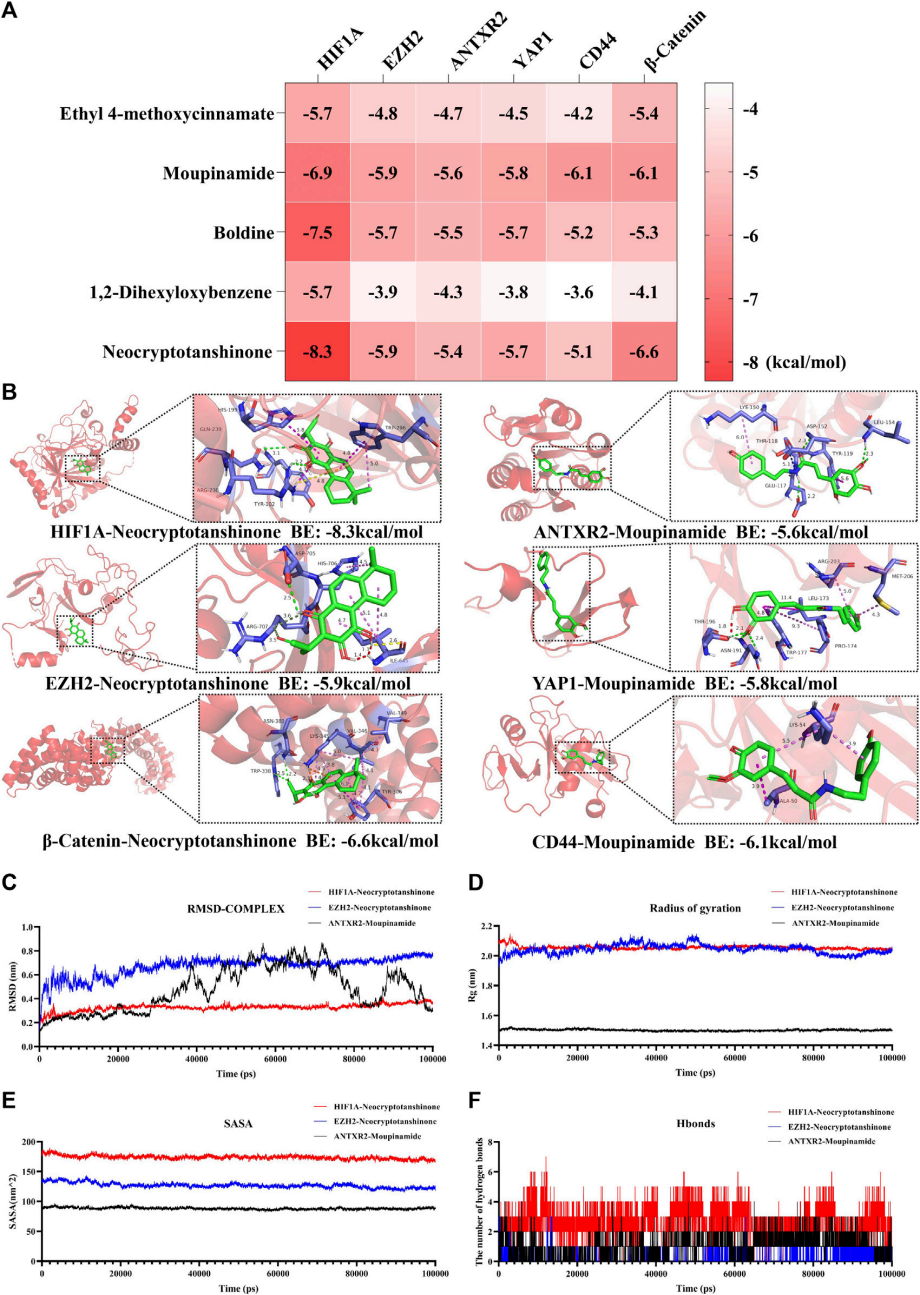
Molecular docking and molecular dynamics simulation results. **(A)** Heat map of molecular docking binding energy. **(B)** Molecular docking patterns with higher binding energy of compounds and pathway-associated targets. Analysis of RMSD **(C)**, Rg **(D)**, SASA **(E)**, and hydrogen bonds **(F)** of HIF-1A-Neocryptotanshinone (red), EZH2-Neocryptotanshinone (blue), and ANTXR2-Moupinamide (black). BE is short for binding energy.

Additionally, the binding energies between the top five core components and the key target were visualized (Figure 9A), and the molecular docking patterns with higher binding energies are also shown (Figure 9B) for better understanding (Jiao et al., 2023). Molecular dynamic simulations are a valuable tool for investigating the dynamics, stability, and structural intricacies of target–ligand complexes. Specifically, the stability of protein–ligand complexes, namely HIF1A-neocryptotanshinone, EZH2-neocryptotanshinone, and ANTXR2-moupinamide, was analyzed in this study. The conformational and structural stabilities of the complexes were assessed using RMSD analysis. The complexes of HIF-1A and neocryptotanshinone and EZH2 and neocryptotanshinone remained stable (Figure 9C), whereas the conformation of the complex ANTXR2–moupinamide showed a slightly larger fluctuation during the simulations, indicating that the small molecules found a position with less energy during the simulation. The radius of gyration (Rg) is an important indicator of the tightness of protein-ligand complexes. The results showed that the three-line segments were relatively stable (Figure 9D), and Rgs was less than 2.15 nm, suggesting that the complexes were stable. The solvent-accessible surface area, a surface area indicator of the protein (Figure 9E), slightly decreased, indicating that the protein gradually tightened. Notably, hydrogen bonds (H-bonds) can form between proteins and ligands (Figure 9F).

## 4 Discussion

EMs remains an incurable condition that necessitates prolonged management, imposing a substantial medical burden and profoundly affecting the physical and mental wellbeing of women (Wang et al., 2022). TCM teaches that EMs is classified under various categories, such as “Zhengjia” and “dysmenorrhea,” and clinical approaches targeting blood stasis in EMs treatment have demonstrated distinctive advantages. An increasing number of studies have proved the efficacy of TCM in alleviating pain, enhancing pregnancy rates, and improving the quality of life with fewer adverse effects (Feng et al., 2023). Consequently, it has emerged as a research hotspot and first-line solution for the long-term management of EMs in China (Ni et al., 2020). LSNYP was formulated by Professor Luo Yuankai, a distinguished specialist in gynecology, and its composition is based on the TCM theory of “Emperor–Minister–Assistant–Messenger.” Within this prescription, Yimucao enhances blood circulation, whereas Zhebeimu disperses phlegm and dissipates masses and is regarded as an emperor herb responsible for treating the main symptoms. Taoren, Danshen, Chuanxiong, Tubiechong, and Shanzha remove blood stasis and promote blood regeneration, assisting the emperor herbs and acting as minister herbs. Puhuang, Wulingzhi, Wuyao, and Yanhusuo provide further assistance by promoting chi and pain relief; they are assistant herbs. Wumei, a messenger herb, removes the malignant flesh and counteracts the excessive promoting effects of other drugs. Combining these herbs resolves blood stasis and disperses masses, promoting qi circulation and relieving pain. However, the current state of research on LSNYP has not been sufficiently advanced. This study combined UPLC-Q/TOF-MS technology and network pharmacology methods to analyze the prototype components and mechanisms of action of LSNYP and provide a theoretical foundation for its utilization.

The intricate chemical composition of TCM prescriptions presents considerable obstacles in investigating the pharmacodynamic material basis. UPLC-Q/TOF-MS technology, known for its exceptional sensitivity, resolution, and accuracy, holds great potential for application in TCM (Liu et al., 2022b). By employing this technique along with drug-likeness screening conditions, we identified 34 absorbed components suitable for network pharmacology analysis. Notably, ethyl 4-methoxycinnamate reduced the levels of IL-1, TNFα, and VEGF and can be helpful for the treatment of inflammatory and angiogenesis-related diseases (Umar et al., 2014). Moupinamide is an alkaloid that plays an important role in reducing free radicals and relieving oxidative stress. Tetrahydropalmatine is often used for pain relief, and the combination of tetrahydropalmatine, ligustrazine, and ferulic acid inhibits the development of endometriotic tissue, decreases E2 levels, and enhances the phagocytic ability of peritoneal macrophages (Gao et al., 2019). Another study (Woo et al., 2019) found that dehydrocostus lactone, a sesquiterpene lactone compound, can reduce the production of PGE2 and neurotrophins and suppress the Akt and NF-kB pathways in human endometriotic cells. Norisoboldine, a primary isoquinoline alkaloid found by Wuyao (Lv et al., 2018), can alleviate colitis in mice and enhance Treg polarization in a hypoxic microenvironment. Vanillin has anti-inflammatory, antibacterial, and antitumor properties. Liu et al.(2023c) found that vanillin decreased the weight and volume of endometriotic tissues through anti-inflammatory and anti-oxidant pathways in a mouse model. Although the parts do not represent the whole, these studies also suggest that LSNYP may treat EMs by regulating hypoxia, anti-inflammation, inhibition of cells, and blood vessel growth.

According to the PPI network analysis results, the core targets included IL-6, EGFR, HIF1A, MMP9, and EZH2. IL-6 is an important inflammatory factor in EMs, and an elevated level of IL-6 in women with EMs reduces the cytolytic activity of NK cells (Kang et al., 2014), activates macrophages, and amplifies angiogenesis (Ayaub et al., 2019; Song et al., 2020). EGFR, which is a tyrosine kinase receptor, can stimulate the formation of blood vessels. EGFR inhibitors can regress endometriotic lesions and reduce MMP-7 activities (Chatterjee et al., 2018). HIF1A, a transcription factor under hypoxic conditions, plays a vital role in the hypoxic mechanism of EMs (Wu et al., 2019), and downregulation of the HIF1A/VEGF pathway can be helpful in restraining the proliferation and invasion of ecESCs (Qin et al., 2023). MMP9, which belongs to the MMP family, can degrade type IV collagen in both epithelial and stromal cells in the surgical tissues of EMs and overexpress MMP-9 (Wang et al., 2018). EZH2, a histone methyltransferase, can suppress the transcription of target genes by catalyzing the trimethylation of lysine 27 on histone 3 (H3K27me3). Loss of EZH2 expression leads to increased downstream gene expression during the decidualization of human endometrial stromal cells (Grimaldi et al., 2011). According to previous reports, depleting EZH2 could upregulate angiotensin-converting enzyme II in human embryonic stem cells (Li et al., 2020) and ANTXR2 in euESCs (Lin et al., 2019). These targets suggested by the PPI network are associated with cell survival and the inflammatory response, which is also consistent with the GO enrichment results; that is, LSNYP can act on multiple functions of the predicted targets, such as response to oxidative stress, cell proliferation, focal adhesion, and transcription factor activity.

Based on the KEGG pathway analysis results, LSNYP may treat EMs by regulating hypoxia-, inflammation-, and cell proliferation-related signaling pathways, such as the HIF-1, AGE–RAGE in diabetic complications, TNF, and MAPK pathways. HIF1A is the active subunit of HIF-1, located in the cytoplasm, under hypoxic microenvironment. HIF1A is transported to the cell nucleus, where it combines with HIF1B to form the HIF (Wilson, 2018). Subsequently, HIF activates downstream genes. Studies have confirmed that HIF1A can regulate estrogen responsiveness, glycolytic metabolism, angiogenesis, cell proliferation, and inflammation in EMs (Zhan et al., 2016). AGE–RAGE binding is associated with oxidative stress and inflammation, and a previous study (Khan et al., 2018) showed that hypoxia induces AGE–RAGE-mediated cancer progression. Sharma et al. (2010) suggested that elevated levels of RAGE, EN-RAGE, and COX-2 are significant factors in the advancement of EMs. The most important angiogenic cytokine in the TNF pathway is TNF-α, which contributes to the generation of inflammatory factors (Sakamoto et al., 2003) and the adhesion of endometriotic stomal cells. A study indicated that resveratrol can acts as a modulator of EMs and inhibit the release of IL-8 induced by TNF-α in ESCs (Khodarahmian et al., 2021). MAPKs, members of the serine/threonine kinase family, have been shown to play a crucial role in the pathogenesis of EMs by regulating processes such as invasion, proliferation, mitosis, angiogenesis, inflammation, and apoptosis (Yoshino et al., 2004; Leconte et al., 2015; Bora and Yaba, 2021; Giacomini et al., 2021).

Recent studies have found that EMs is a complex clinical syndrome of gene–gene and gene–microenvironment interactions, and the accumulation of HIF1A in hypoxic conditions is the key driving factor of EMs pathogenesis (Wu et al., 2019). To provide further evidence for the application of LSNYP, combined with the results of network pharmacology and current related research, we chose the HIF1A/EZH2/ANTXR2 pathway for experiments and molecular docking verification. HIF1A can inhibit EZH2, and abnormal methylation modifications result in an increase in downstream ANTXR2 expression in EMs (Lin et al., 2019). ANTXR2, also known as capillary morphogenesis gene 2 (CMG2), is involved in tumor angiogenesis (Ma et al., 2023), and activating ANTXR2 could promote the proliferation and vitality of ESCs (Liu et al., 2023b). YAP1, CD44, and β-catenin are the downstream proteins of ANTXR2. HIF1A expression is positively related to YAP1 in ectopic endometrial tissue. When suppressing the YAP1 function, key biological processes involved in EMs development, such as steroidogenesis, inflammation, and migration, are reduced (Lin et al., 2017). CD44 is a transmembrane glycoprotein, and increased CD44 splice variant expression in the eutopic endometrium makes it easier for shed endometrial cells to adhere to the abdominal cavity (Griffith et al., 2010; Knudtson et al., 2016). β-catenin is associated with the invasive phenotype of eutopic endometrium. When the β-catenin gene was silenced with siRNA, the expression of its downstream target genes VEGF and MMP-9 was significantly inhibited (Liu et al., 2016). Therefore, inhibiting the HIF1A/EZH2/ANTXR2 pathway may help treat EMs. In these cell phenotype experiments, the results indicated that LSNYP suppressed the viability of ecESCs in a concentration-and time-dependent manner and that the adhesion, migration, and invasion abilities of ecESCs were reduced after 72 h of intervention with H-LSNYP, indicating that LSNYP can inhibit the growth of ecESCs. Western blotting results showed that LSNYP treatment reduced the expression levels of HIF1A, ANTXR2, YAP1, CD44, and β-catenin, and increased EZH2 expression in ecESCs. When the HIF1A knockdown vector was used to treat cells with LSNYP, the inhibitory effect of LSNYP on this pathway was more apparent. In the animal experiments, LSNYP ameliorated pathological injury and inflammatory responses, regulated the expression of genes and proteins related to HIF1A/EZH2/ANTXR2 pathway in endometriotic tissues. Additionally, the molecular docking and molecular dynamics simulation results showed that moupinamide and other core components are stably bound to the HIF1A, EZH2, ANTXR2, YAP1, CD44, or β-catenin. These findings suggest that this prescription may exert therapeutic effects by inhibiting the HIF1A/EZH2/ANTXR2 pathway in EMs.

This study has some limitations. First, identifying serum components and targets from self-built or existing databases may not be sufficiently comprehensive, and standard products should be added for quantitative analysis. Second, due to the limitation of time constraints and experimental funding, the sample size of the animal experiment was low, which may produce a bias in the expression of the results. Future studies expand the sample size and add other detection methods and indicators.

In summary, the mutual confirmation of serum pharmacochemistry, network pharmacology, and experiments enabled a preliminary exploration of the pharmacological mechanism of LSNYP in EMs, which may be related to the regulation of hypoxia and suppression of the HIF1A/EZH2/ANTXR2 pathway, providing theoretical evidence for the clinical application of LSNYP. The TCM formula, LSNYP, shows potential as an effective agent for preventing and treating EMs.

## Data Availability

The original contributions presented in the study are included in the article/Supplementary Material, further inquiries can be directed to the corresponding authors.
