# Supplementary material for: Potential mechanism of Luoshi Neiyi prescription in endometriosis based on serum pharmacochemistry and network pharmacology
Source: Front Pharmacol. 2024 Jul 29;15:1395160. doi: 10.3389/fphar.2024.1395160 (PMC11317381; doi:10.3389/fphar.2024.1395160)
Supplement: Supplementary file 1 [file DataSheet2.PDF]

Table S2: 45 components were identified in LSNYP-containing serum

NO	RT (min)	Adduct ion	Theoretical	Experimental m/z	Error (ppm)	MS^2	Molecular formula	Component name	Categories
1	4.05	+HCOO	520.1661	520.1674	2.5	520.1674, 474.1585, 318.0209, 161.0349	$C_{20}H_{29}NO_{12}$	Mandelic amide-β-gentiobioside	saponin
2	5.88	+H	139.0390	139.0391	0.7	93.0391, 65.0385	$C_7H_6O_3$	Protocatechualdehyde	phenolic acid
3	6.26	+H	139.0390	139.0392	1.4	93.0392, 65.0385	$C_7H_6O_3$	p-Hydroxybenzoic	phenolic acid
4	7.87	+HCOO	405.1391	405.1388	-0.7	405.1388, 295.0856, 96.6467	$C_{16}H_{24}O_9$	Nikoenoside	saponin
5	8.19	+HCOO	669.1661	669.1662	0.1	669.1662, 583.1903, 508.0599, 323.0911, 211.9923, 132.0347	$C_{28}H_{32}O_{16}$	Isorhamnetin-3-O-neohesperidoside	flavonoid
6	8.23	+H	458.1657	458.1663	1.3	458.1663,296.1127, 278.1028, 158.0599, 134.0598, 97.0283	$C_{20}H_{27}NO_{11}$	Amygdalin	saponin
7	8.47	-H	475.1457	475.1448	-1.9	475.1448, 389.0663, 323.1274, 269.0941, 161.0351, 113.0141	$C_{20}H_{28}O_{13}$	Mandelic acid-β-gentiobioside	flavonoid
8	8.66	+H	314.1387	314.1385	-0.6	314.1385, 298.1068, 265.0844, 237.0896, 205.0649, 177.0706	$C_{18}H_{19}NO_4$	Norbracteoline	alkaloid
9	9.86	+H	153.0546	153.0549	2.0	153.0549, 110.0358, 93.0328, 65.0384	$C_8H_8O_3$	Vanillin	phenolic acid
10	10.81	+HCOO	427.2115	427.2135	4.7	427.2134, 379.1721, 311.0989, 149.0709, 101.0153	$C_{24}H_{30}O_4$	Senkyunolide P	phthalide
11	10.92	+H	328.1543	328.1548	1.5	328.1548, 297.1111, 265.0856, 178.0859, 163.0625, 151.0752	$C_{19}H_{21}NO_4$	Stepholidine	alkaloid
12	11.09	-H	313.1445	313.1440	-1.6	313.1440, 267.1527, 227.1282, 195.0071, 114.0190	$C_{19}H_{22}O_4$	Neocryptotanshinone	flavonoid
13	11.45	+H	448.1966	448.1972	1.3	448.1972, 430.1860, 421.1750, 269.1161, 175.0750, 107.0488	$C_{23}H_{29}NO_8$	N-Methylcoclaurine-7-O-β-D-glucopyranoside	saponin
14	11.52	-H	609.1461	609.1488	4.4	609.1488, 581.2239, 417.1152, 300.0193	$C_{26}H_{28}O_{14}$	Apigenin 6-C-arabinoside 8-C-glucoside	flavonoid
15	11.74	+H	314.1387	314.1391	1.3	314.1391, 297.1109, 265.0858, 237.0908, 205.0646, 194.0724, 177.0693	$C_{18}H_{19}NO_4$	Norisoboldine	alkaloid
16	12.17	+H	328.1543	328.1557	4.3	328.1557, 297.1116, 282.0885, 265.0858, 239.0702, 205.0646, 177.0698, 165.0697	$C_{19}H_{21}NO_4$	Boldine	alkaloid
17	12.56	+H	328.1543	328.1546	0.9	328.1546, 296.1041, 282.0869, 265.0857, 253.0857, 165.0697	$C_{19}H_{21}NO_4$	Isoboldine	alkaloid
18	12.66	-H	491.1559	491.1540	-3.9	491.1540, 429.1499, 315.1153, 253.1141, 113.0147	$C_{24}H_{28}O_{11}$	Macrophylloside A	saponin
19	12.94	+H	373.2221	373.2218	-0.8	373.2218, 208.0974, 193.1646, 175.1476, 133.1008, 65.0399	$C_{19}H_{32}O_7$	Byzantionoside B	saponin
20	13.05	+H	356.1856	356.1851	-1.4	356.1851, 192.1019, 177.0781	$C_{21}H_{25}NO_4$	Rotundine	alkaloid
21	13.98	+H	356.1856	356.1863	2.0	356.1863, 340.1548, 204.1014, 191.0939, 178.0865, 163.0631, 151.0754, 119.0494	$C_{21}H_{25}NO_4$	Tetrahydropalmatine	alkaloid

22	14.35	+H	352.1543	352.1545	0.6	352.1545, 336.1227, 290.0927, 275.0698, 150.0670	$C_{21}H_{21}NO_4$	13-Methyl-dehydrocorydalmine	alkaloid
23	14.98	+H	352.1543	352.1540	-0.9	352.1540, 337.1308, 322.1079, 306.0761, 278.0803	$C_{21}H_{21}NO_4$	Dehydrocorydaline	alkaloid
24	15.12	+H	207.1016	207.1017	0.5	207.1017, 178.0775, 133.0639, 91.0541, 77.0378	$C_{12}H_{14}O_3$	Senkyunolide F	phthalide
25	15.67	+H	207.1016	207.1023	3.4	207.1023, 133.0644, 115.0544, 105.0697, 91.0543, 77.0385	$C_{12}H_{14}O_3$	Ethyl 4-methoxycinnamate	phenolic acid
26	16.18	+H	352.1549	352.1544	-1.4	352.1544, 336.1232, 308.1276, 293.1049, 279.0898	$C_{21}H_{22}NO_4^+$	Palmatine	alkaloid
27	16.52	+H	314.1387	314.1392	1.6	314.1392, 177.0545, 149.0598, 145.0285, 121.0648, 89.0385, 77.0387	$C_{18}H_{19}NO_4$	Moupinamide	alkaloid
28	16.95	+HCOO	507.2436	507.2429	-1.4	507.2428, 461.2366, 315.1729, 161.0349	$C_{22}H_{38}O_{10}$	Linalyl rutinoside	terpenoid
29	18.84	+H	231.1374	231.1384	4.3	231.1386, 185.1316, 142.0778, 119.0856, 105.0703, 81.0699, 79.0544	$C_{15}H_{18}O_2$	Dehydrocostuslactone	phthalide
30	20.74	+H	231.1380	231.1384	1.7	231.1384, 213.1276, 187.1116, 141.0706, 128.0619, 91.0547, 65.0399	$C_{15}H_{18}O_2$	Shizukanolide A	phthalide
31	20.83	+H	293.2111	293.2113	0.7	293.2113, 275.1994, 163.1123, 119.0852, 105.0703, 91.0543	$C_{18}H_{28}O_3$	17β-Hydroxy--2-oxa-5α-androstan-3-one	steroid
32	20.84	-H	309.1191	309.1194	1.0	309.1194, 294.0947, 195.0207, 150.9937, 96.9500	$C_{12}H_{22}O_9$	Neoeriocitrin	flavonoid
33	21.61	+H	189.0910	189.0914	2.1	189.0914, 133.0286, 128.0622, 105.0338, 77.0386	$C_{12}H_{12}O_2$	n-Butylidenephthalide	phthalide
34	22.04	+H	295.2268	295.2273	1.7	295.2773, 277.2184, 151.1121, 105.0697, 91.0544, 79.0541	$C_{18}H_{30}O_3$	9-Carbonyl-10E,12Z-octadecadienoic acid	aliphatic acid
35	23.09	+H	231.1380	231.1385	2.2	231.1385, 213.1264, 183.0844, 175.0756, 157.0648, 128.0623, 67.0544	$C_{15}H_{18}O_2$	Lindenenol	terpenoid
36	23.91	+H	247.1329	247.1332	1.2	247.1332, 201.1276, 159.0805, 143.0857, 129.0699, 115.0544, 91.0543	$C_{15}H_{18}O_3$	Hydroxylinderstrenolide	terpenoid
37	24.27	-H	253.1346	253.1357	4.3	253.1357, 209.1448	$C_{16}H_{18}N_2O$	Lysergol	steroid
38	26.16	+CH3COO	475.2326	475.2315	-2.3	475.2315, 299.1939, 267.1862, 175.0114, 113.0114	$C_{24}H_{32}O_6$	Deoxyschizandrin	lignan
39	26.97	+H	279.2319	279.2326	2.5	279.2326, 219.0580, 201.0471, 173.0535	$C_{18}H_{30}O_2$	1-(4-nonylphenoxy)-2-propanol	phenolic acid
40	27.22	+H	279.2319	279.2329	3.6	279.2329, 237.9929, 201.0467, 173.0509, 149.0235	$C_{18}H_{30}O_2$	1,2-Dihexyloxybenzene	phthalide
41	27.83	+H	309.1121	309.1126	1.6	309.1126, 265.1220, 223.0752, 165.0697	$C_{19}H_{16}O_4$	Tanshinoldehyde	saponin
42	28.78	+H	297.1485	297.1494	3.0	297.1494, 279.1382, 237.0912, 165.0697, 141.0697, 128.0619	$C_{19}H_{20}O_3$	Cryptotanshinone	quinone

43	29.55	-H	291.1027	291.1015	-4.1	291.1015, 211.1413, 111.9191, 80.9556	$C_{19}H_{16}O_3$	1,2-Dihydrotanshinone IIA	quinone
44	31.55	+H	297.1485	297.1497	4.0	297.1497, 282.1252, 279.1379, 268.1095, 254.0939, 249.0913, 239.0704, 141.0697	$C_{19}H_{20}O_3$	Isocryptotanshinone	saponin
45	31.99	-H	595.2913	595.2918	0.8	595.2918, 315.0409, 279.2245, 241.0026, 152.9853	$C_{34}H_{44}O_9$	Salannin	terpenoid
