# Supplementary material for: Potential mechanism of Luoshi Neiyi prescription in endometriosis based on serum pharmacochemistry and network pharmacology
Source: Front Pharmacol. 2024 Jul 29;15:1395160. doi: 10.3389/fphar.2024.1395160 (PMC11317381; doi:10.3389/fphar.2024.1395160)
Supplement: Supplementary file 2 [file DataSheet4.PDF]

Table S4: 1119 EMs-related targets

ENDO1	CDKN2B-	H19	MIR424	MIR145	MIR141
WNT4	MIR125A	MIR34C	MIR126	MIR200B	MIR29C
MIR20A	MIR100	MIR223	MIR142	MIR143	MIR200A
MIR196B	MIR150	MIR99A	MIR99B	RN7SL7P	CYP19A1
ESR1	PGR	VEGFA	ESR2	IL1B	IL6
GSTM1	TP53	HOXA10	PTGS2	GNRH1	MMP2
PTEN	CCL2	CXCL8	CYP17A1	MUC16	CYP1A1
TNF	MMP3	NR5A1	HSD17B1	MMP1	IL1R2
EMSLR	EMX2	TIMP2	KDR	CCL5	NAT2
HNF1B	HGF	CYP1B1	IL10	MMP7	MMP9
AHSG	PAEP	FSHR	MIF	LIF	MIR199A1
ARID1A	ARNT	ENO1	RXFP1	STX5	PLAU
STAR	IGFBP1	CXCL5	STS	GNRHR	KRAS
MIR17	MIR200C	TIMP1	MIR449B	CALB2	CGA
GSTT1	FLT1	AMH	CCN1	NR2F2	CTNNB1
MIR183	PIK3CA	MIR21	EBAG9	BCL2	LEFTY2
MME	CDKN2A	NGF	COMT	ICAM1	BDNF-AS
TGFB1	LINC00339	GPER1	SPP1	IFNG	MIR451A
UBOX5-AS1	THOC6	HSD17B2	LEP	MIR139	CDH1
IL1A	HLA-DRB1	PAX2	AHR	MIR361	PPP2R1A
FAS	MIR148A	HLA-B	ENSG00000285959	VIM	IL1R1
MIR221	AR	TNFRSF1B	AHRR	ITGB3	PTPN22
HOXA11	IL2	FBN1	CD44	GSTP1	EGF
IGF1	LTA	HIF1A	AKT1	MAP2K7	TMX2-CTNND1
SERPINE1	IL18	MIR510	GC	IL4	MIR125B1
CDKN1B	PPARG	POLE	IL33	OXTR	MIR320A
CHL1-AS1	FASLG	BAX	CCR1	TLR4	ACE
LHB	LOC108491836	XRCC1	PRL	KRT7	EGFR
GREB1	GALT	HLA-A	IL1RN	FN1	TLN1
MALAT1	CXCL12	MIRLET7B	SNAI1	CYP2C19	NFKB1
IL15	NTRK2	VEZT	IL37	ANXA2	CSF1
CDKN1A	KLF9	MIR9-1	MIR25	IL17A	VDR
HLA-C	C5	ERLNC1	NOS3	MIR29A	MIR182
VCAM1	MIRLET7C	TNFRSF1A	CCND1	MIR483	MIR34A
MUC1	MIR146B	MIRLET7D	HLA-G	CTLA4	TNFRSF11B
HOXA11-AS	FGF2	SOD2-OT1	IGF1R	BMP6	MLH1
MIR22	LOC110386951	FGFR2	SIRT1	IL16	FOXP3
MCM2	MCM7	APEX1	CDKN2A-DT	HP	HPSE
RELA	MIR23A	CRH	GNRHR2	MIR92A1	CXCL1
PCNA	MAPK1	BRCA1	IL11	LEPR	FCRL3

WT1	BDNF	DNMT1	CLDN4	ANGPT2	OGG1
BIRC5	BSG	FKBP4	HLA-DQA1	LINC02605	IGFBP3
MIR31	BRCA2	MIR210	BRAF	EZH2	PAX8
MKI67	MAPK8IP1	AOAH	MIR23B	MST1	UCN
TERT	ERBB2	HMGB1	ITGB1	FST	PROK1
MPO	STAT3	YAP1	ACP1	LTF	MIR191
HDAC1	SNORD15A	MET	AXL	STAT4	CXCR4
GHRL	MIR24-1	TRAF1	CXCR2	EZR	MIR423
NAPA-AS1	FOS	IGF2	NR3C1	FSHB	MIR574
EED	CHL1-AS2	TTTY8	TTTY11	TTTY22	IL12B
CCL11	PDCD1	IL32	MIR215	IL6R	INHBA
SMAD3	FGF1	NOTCH1	SMAD4	VEGFC	MIR214
CDH2	C3	PHB1	CXCL10	NRP1	MMP14
TRPV1	PTGER4	MMP13	PGRMC1	DNMT3B	CXCR1
MST1R	SERPINF1	BCL6	HNRNPA2B1	DNMT3A	MIRLET7A1
CCR5	KRT19	XRCC4	CERNA3	THBS1	ACTC1
TNFSF13B	NPY	ADIPOQ	CFL1	TWIST1	RHOU
MIR106B	MIR370	MIR205	MIR106A	HLA-DPB1	SERPINA6
TET2	TIMP3	ADAM17	GAS5	CSF2	ANGPT1
MIR125B2	MIR26B	MIR98	CCL21	FOXP1	MIR331
MIR486-1	MTOR	LGALS3	FGFR1	WFDC2	TDGF1
KRT20	CD40	MIR199B	GNPTAB	MAPK3	IL13
MIR101-1	BGLAP	MIR381	MIR610	IL1RAP	CLDN3
IL27	HMOX1	AKR1C3	SNORD44	MIR27B	MIRLET7F1
RNU6-1	RNU6-2	RNU6-7	RNU6-8	RNU6-9	RNU6-1-001
RNU6-1-002	RNU6-1-003	RNU6-1-004	MIR124-1	DES	BCL2L1
GDF15	ARSH	LINC01672	NTRK1	ERCC2	RETN
MTHFR	IL1RAPL2	TET1	MIR199A2	MIR28	MIR30A
SOD1	CD40LG	IL2RB	DRD2	TAGLN	MIR10A
MIR16-1	MIR18A	MIR103A1	MIR202	MICB	MICA
MIR1915HG	SMAD2	PPARGC1A	ANG	IL5	VIP
PELATON	ENSG00000232995	TNFSF10	TNFRSF10B	CCNB1	GATA6
SULT1E1	NODAL	CD82	PDE1C	RND3	SORCS1
ID4	CSMD1	ITPK1	RNF144B	ZBTB40	KSR2
MOAP1	RBM43	OR1D2	OR1G1	HNRNPA3P1	LNC-LBCS
MCPH1-DT	MYC	TH	SERPINC1	MIR154	F2RL1
CREB1	POU5F1	KISS1	MUC4	APOE	RHOA
ITGA6	IDO1	ADM	MIR196A1	MIR144	METTL3
DEFA1	LMNB1	NGFR	CYP2E1	NAT1	RUNX3
MIR185	MIR222	S100A4	CCDC144NL-AS1	AGTR1	SRC
PDGFA	NFE2L3	DEFA10P	MIR27A	AMHR2	COX5A
MIR24-2	PGR-AS1	CYP24A1	YBX1	BAD	TYMP
MIR3685	MIR149	MIR146A	TEK	JUN	IRF5
CCL25	MEG3	F2	MIR130A	TXN	TXNIP

PTK2	TACR1	HRAS	MIR155	MIR15A	MIR506
CDC42	CDKN2B	PTPRD	CKAP2L	PWAR1	IRS2
SEMA3C	MIR133B	EPCAM	SHBG	CCL3	ITGAV
EIF4EBP1	KITLG	HMGA2	CCL19	CAT	FGA
CDH3	NRIP1	ELANE	ENG	ANXA1	SYP
RARRES2	PCAT1	MIR92A2	NCOR2	GREM1	AKAP13
IL1RL1	TGFB1I1	PGRMC2	PCDH17	UCA1	MIRLET7F2
CALCA	CD200	MIR33B	ADA	NLRP3	CLU
STIP1	CXCR3	ID2	IGFBP4	PF4	STOML2
KLF11	XIST	ENSG00000277577	ZEB1	IFNA2	EPOR
ENO2	OPRM1	PARK7	CCR2	CHI3L1	CLOCK
ERCC5	LGR5	MDK	TRIM24	XRCC3	ACKR3
EPO	PTGES	LGALS9	ERVW-1	DHRS4-AS1	MIRLET7G
RPL5	DDX6	METAP2	XRCC5	CBX3	DDX39B
HNRNPF	SEPTIN9	SNRPD2	NR1H2	NUMB	PRDM1
PAPPA	MAP1LC3A	PDCD6	STUB1	MIR122	GJA1
TSLP	U2AF1	MIR127	RAF1	EDNRA	STK4
CD79A	CDK1	EDN1	PGM1	DCN	CASP9
COL18A1	CRP	FEN1	MMP12	ROCK2	VCAN
CD274	SOX2	SLC18A3	HLA-DQB1	MMP24	CCR9
HNRNPC	TET3	FSTL3	S100A13	HOTAIR	MIR203A
LINC01116	TF	MIR532	ENSG00000277966	ENSG00000278592	ENSG00000273961
ENSG00000276496	ENSG00000278020	ENSG00000278334	NECTIN4	TLR2	EIF4E
GDF9	USF2	TP63	MSC-AS1	SMAD5-AS1	UCHL1
NME1	PLK1	AGER	ACVR2A	PRDX6	HTRA1
SNCG	CNTF	EIF3E	KIR3DS1	LINC01133	KIR2DS5
TCF21	IL6ST	ROCK1	CEBPA	CD69	RPS23
NDUFA13	CX3CL1	TMOD3	MIR3926-1	CTSD	STAT6
IGFBP7	ALOX15	EPHX1	EPRS1	USP10	RACK1
USF1	RPS27A	CCR8	DNMT3L	ABCG2	MIR29B1
MIR340	KRT8	ETS1	TGFBR1	NFKBIA	MIR93
TEX41	CTSB	HSPD1	ABCC4	C9	RMRP
CCNA2	SNHG4	KIR3DL1	KIR2DL3	SHC1	LCN2
TLR3	CDK6	CSNK1A1	SLC6A4	HMGA1	PHB2
MIR101-2	CDH12	SORBS1	ITGBL1	IGHM	SPINK1
PTP4A3	KLRK1	MSI1	PDYN	AIF1	TRA-TGC7-1
TRA-TGC5-1	LDHA	GH1	HSD3B2	MIR503	AGR2
S100P	MAPK14	EIF2AK3	SOD2	ERN1	INHA
MIR542	KMT2A	SIRT3	LHCGR	SDC1	HIF1A-AS2
LINC01541	CD163	AFM	LAMC2	NRP2	AKR1C1
TFF3	MIR26A1	MIR15B	MIR26A2	MIR425	MIR103A2
MIR16-2	MIR95	PAK1	NOS2	SERPINA1	LOXL4
TNFRSF21	MUC2	SNORD75	SRD5A1	S100A6	RPLP1

LOC108863620	L1CAM	ULBP2	SP2-AS1	MIR4457	CYP3A4
GLB1	NR4A1	BECN1	LIPC	DROSHA	IGF2BP3
ELAVL1	GHRH	MIR493HG	ENSG00000276919	TPM3	CPB2
EFNA1	CALD1	NPTX2	PIBF1	STAT1	PAK4
XDH	SERPINB2	RNY3	GPX4	CDH13	COPS5
FJX1	ALPP	NCOA1	CSF1R	DAPK1	TNFRSF10A
STC1	TSPAN1	BCAR3	CCDC80	ADAMTS9-AS2	RASSF1-AS1
CD36	FOXO1	UGT1A1	GATA3	NCAM1	KRT17
APOBEC3B	APOBEC3A	LEPQTL1	MIR10B	MIMT1	JAK1
NFE2L2	PRKCB	PON1	AKR1B1	FTO	HSF1
TGFBR3	GCLC	IL12RB2	OSM	JUNB	PRX
NINJ1	NTN4	CAMP	NANOG	CYRIB	MT-CO2
DIRAS3	JPX	ENSG00000274430	ENSG00000276784	LOC110408762	BIRC2
BAK1	CSF3	SNHG5	MIR363	TYK2	ABL1
NT5E	TGFB2	STK11	RHEB	RPS6KB1	TSC2
ATF6	NF1	TGFB3	AXIN1	F3	CCR7
STMN1	DYSF	CXCL11	CD1A	CD83	HAS2
PIAS3	RBP1	CCL8	CXCL9	CRISPLD2	HOXB4
DEFA3	YTHDF2	KIR2DL1	HLA-DRB4	MIR196A2	MIR497
MIR675	MIR29B2	MIR629	MIR885	PAX8-AS1	MIR543
CRK	GAS6	MIR4748	ALDH2	GSK3B	DICER1
KCNQ2	CDC25A	CTCF	CRYAB	FOXA2	GRB2
KISS1R	PROM1	PTGS1	ADAR	PPM1A	S1PR1
SLIT2	AVP	CD47	CHIT1	CORO1A	EXTL3
FHIT	PGF	PRDX5	SEMA3F	RHOC	CHD5
ETS2	NUP210	PTN	STING1	VEGFD	ANXA4
CCDC22	FOXL2	GRHL2	MAS1	NLRC5	TAGLN2
UGT2B28	INTU	RNH1	MUC17	TRIM59	USP17L2
MIR135A1	MIR135A2	MIR1271	MIR1260A	MIR4521	MIR4476
MIR548L	MIR3141	MIR3182	MIR4508	LOC110386948	KRT18
PRKG1	KLK3	SRA1	SNHG17	MEIS1	CHUK
AGT	CASP1	SLC11A2	TAF1	ALOX5	F5
PLA2G6	ACTA2	CCN2	CETP	HPGD	PRDX2
RBP4	BGN	CIITA	MAF	MFGE8	RORC
AQP9	BLMH	CAPN5	HSD17B7	MSLN	SMOC2
UGCG	CREB3L1	ID1	LUM	PRELP	SMPD3
TNFRSF6B	ILKAP	MYH8	SLPI	IL34	MRC1
SGMS1	TRERF1	KIR2DS4	LINC00473	MIR32	MIR499A
CARMN	MIR1-1	MIR30D	MIR1-2	MIR449C	TTTY14
MIR500A	MIR502	MIR514A1	MIR4497	MIR514A2	MIR514A3
TTTY23B	TTTY9B	TTTY23	TTTY21	MIR499B	MIR6795

TRN-GTT2-6	ADH5P2	TRN-GTT2-1	TRN-GTT2-5	UBA52P1	TRN-GTT2-3
TRN-GTT2-4	TRN-GTT2-2	TRN-GTT2-7	TTTY23-001	TTTY9A-001	TRN-GTT2-8
MIR187	IDH1	CBR1	EEF1A1	FABP4	PRPF8
EEF1D	RPL8	DUSP2	FIP1L1	STOM	ALYREF
HNRNPH2	NAT10	SYNCRIP	GCN1	CASP8	HDAC2
AURKA	AURKB	LAMB1	CD55	FKBP5	GATA2
ITGA5	LIMK1	PLG	TNC	KNG1	CSF3R
CNR1	FCGR3A	ISG15	ITGA2	ANTXR2	BDKRB2
CLCN3	HAVCR2	HMOX2	LAMA1	MYH11	SIRPA
NR5A2	SDC4	SNAI2	CD63	HAMP	LAMC1
P2RX3	SELE	BDKRB1	SLIT3	TFDP1	NPSR1
TBX4	IL17RE	NCR1	PEMT	RARRES1	RECK
BNC2	CXCL6	IL25	IPO13	NRN1	CHCHD2
KLF12	PROKR1	MRPL28	TTC39B	IGF2-AS	TRB
MIR34B	MIRLET7I	DNM3OS	MIR328	MIRLET7A2	MIRLET7A3
AFAP1-AS1	MIR320B1	MIR320C1	MIR320B2	MIR320D1	MIR4284
MAP4K3-DT	MIR320C2	MIR320D2	MIR4710	MIR5187	MIR4454
MIR4714	MIR5193	MIR3152	MIR3667	MIR3680-1	MIR3680-2
MIR195	MIR342	MAPK9			
