# Supplementary material for: Potential mechanism of Luoshi Neiyi prescription in endometriosis based on serum pharmacochemistry and network pharmacology
Source: Front Pharmacol. 2024 Jul 29;15:1395160. doi: 10.3389/fphar.2024.1395160 (PMC11317381; doi:10.3389/fphar.2024.1395160)
Supplement: Supplementary file 3 [file DataSheet6.PDF]

Table S6: The binding energy (kcal/mol)

Degree	Components	HIF1A	EZH2	ANTXR2	YAP1	CD44	β-catenin
46	Ethyl 4-methoxycinnamate	-5.7	-4.8	-4.7	-4.5	-4.2	-5.4
46	Moupinamide	-6.9	-5.9	-5.6	-5.8	-6.1	-6.1
34	Boldine	-7.5	-5.7	-5.5	-5.7	-5.2	-5.3
33	1,2-Dihexyloxybenzene	-5.7	-3.9	-4.3	-3.8	-3.6	-4.1
33	Neocryptotanshinone	-8.3	-5.9	-5.4	-5.7	-5.1	-6.6
32	1-(4-nonylphenoxy)-2-propanol	-5.8	-4.9	-4.4	-3.9	-4.2	-5.1
31	Deoxyschizandrin	-7	-5.5	-4.1	-5.1	-4.9	-5
30	Isoboldine	-8.6	-6.1	-6.5	-7	-5.7	-6.3
29	Senkyunolide F	-6.1	-5	-4.6	-5.6	-5.9	-5.7
27	Palmatine	-7.7	-6.1	-5.5	-4.6	-5.1	-5.5
26	Cryptotanshinone	-8.7	-6.7	-5.3	-6.8	-5.8	-6.7
22	Tetrahydropalmatine	-8.1	-5.9	-4.8	-5.8	-5	-6.2
22	17β-Hydroxy--2-oxa-5α-androstan-3-one	-7.4	-5.8	-4.8	-6.1	-5.2	-6.6
21	Dehydrocorydaline	-7.5	-6.2	-5.7	-5.7	-5	-5.9
21	Rotundine	-8.2	-6.2	-5.2	-6.1	-5.4	-6.6
21	Stepholidine	-8.2	-6.8	-5	-6.1	-5.9	-6.6
20	Norisoboldine	-8.1	-5.9	-5.5	-6.2	-5.7	-6
19	13-Methyl-dehydrocorydalmine	-8.1	-5.9	-5.4	-5.4	-5.6	-6.3
18	Macrophylloside A	-9.3	-6.6	-5.9	-6.4	-5.7	-6.7
18	Senkyunolide P	-7.5	-6.4	-6	-6.3	-6	-7.5
15	Byzantionoside B	-8.5	-6.1	-5.6	-5.6	-5.8	-6
15	n-Butylidenephthalide	-7.2	-5.5	-4.8	-5.1	-5.4	-5.5
15	Norbracteoline	-8.5	-6.2	-5.6	-5.9	-5.8	-5.7
13	Tanshinoldehyde	-8.7	-7.4	-6	-6.9	-6.2	-6.6
12	Vanillin	-5.6	-4	-4.1	-4.5	-4.8	-4.2
11	Lysergol	-6.8	-5.8	-5.1	-5.8	-5.9	-6.1
10	Dehydrocostuslactone	-7.9	-5.5	-5	-5.5	-5.5	-5.9
10	Nikoenoside	-7	-5.9	-5.8	-5.6	-5	-5.6
9	Hydroxylinderstrenolide	-7.1	-5.9	-5.2	-6	-5.8	-6.1
9	p-Hydroxybenzoic acid	-5.5	-4.7	-4.2	-4.6	-4.8	-4.2
8	Protocatechualdehyde	-5.5	-4.1	-4.2	-4.8	-4.9	-4.2
5	Lindenenol	-6.9	-5.1	-4.5	-5.4	-5.3	-5.9
3	Isocryptotanshinone	-9	-6.6	-5.6	-6.7	-5.7	-6.8
1	Shizukanolide A	-7.1	-5.5	-4.8	-6.6	-5.5	-6.5
