# Supplementary material for: Potential mechanism of Luoshi Neiyi prescription in endometriosis based on serum pharmacochemistry and network pharmacology
Source: Front Pharmacol. 2024 Jul 29;15:1395160. doi: 10.3389/fphar.2024.1395160 (PMC11317381; doi:10.3389/fphar.2024.1395160)
Supplement: Supplementary file 4 [file DataSheet3.PDF]

Table S3: 1149 Components-related targets

ACER2	ACACB	ADA	ADRB1	ADRB2	ADRB3	CALM1
CNR1	CNR2	ENPP2	FAAH1	FFAR1	FUT7	GGPPS
GP174	GPR88	GTR1	HRH2	KCNK2	LKHA4	LPAR1
LPAR2	LPAR3	LOX5	LX15B	MDHC	MGLL	MMP25
NR5A2	OXER1	P2Y10	PA24B	PA24A	PA2G5	PA2GA
PA2GX	PAR10	PP1G	PPARA	PPARG	PTPRO	RARA
RARB	S100B	S1PR2	S1PR4	S1PR3	S1PR5	S22A2
S22A3	S47A2	SAT1	S1PR1	SCNNA	SGMR1	SPHK2
SPHK1	STF1	STS	TAOK3	THA	THB	TLR8
TRPM2	UNG	ALOX5AP	SLC6A2	AR	PTGS2	SLC6A4
VDR	HSD11B1	MAPK14	IGF1R	HTR2A	IL6ST	PRKCD
PRKCG	PRKCA	PRKCB	PRKCE	PRKCH	PRKCQ	ESR1
ADRA2C	SHBG	ABL1	SMO	F2R	HSP90AA1	PDE10A
ACHE	PYGL	CHRM1	GRM1	BRAF	ADORA3	FAAH
ICMT	MERTK	MAPK1	PDE5A	SLC6A3	MTOR	PIK3CA
MDM2	CYP24A1	HTR2C	PDE2A	PLK1	RASGRP1	C5AR1
KCNA5	CDC7	KCNH2	GABRB3	GABRA2	PIM1	SORD
PIM2	HMGCR	SMYD2	MAPK13	MAPK8	METAP1	P2RX7
SCD	MDM4	CACNA2D1	CACNA2D2	NR3C1	CRHR1	PRCP
ACACA	PRF1	EPHX1	LIMK2	MMP14	ALOX5	TTR
AVPR1A	ADORA1	ADORA2B	AMPD3	GYS1	CCNB3	CDK2
CCND1	AXL	TYRO3	MAP3K14	PTGES	MAPK10	P2RX3
RBP4	CDK5R1	SYK	5HT1B	5HT1A	5HT1D	ACM1
ACM2	ACM4	ACM5	ACM3	ADA1A	ADA1B	ADA1D
ALBU	ASAH1	CAH3	CD14	CP2CJ	DGLA	DHB3
DPOLL	DPOLM	DYN1	DYN2	EBP	EST1	EST2
GBRA6	GPR34	HYEP	KCNA3	LT4R2	LT4R1	LY96
MPEG1	MPIP1	NCEH1	NLRP1	NMDE1	PA2GD	POLH
POLK	RAD52	SOAT1	TLR4	LTA4H	MC4R	RORC
RORB	RORA	GRM5	VCP	CYP11B1	CYP11B2	CYP19A1
DUSP3	PIK3CB	GABRA1	PTGS1	GRM2	AVPR2	NPY5R
TSPO	PLA2G2A	PGR	ADORA2A	EP300	PFKFB3	HCRTR2
HCRTR1	CXCR2	CYP17A1	FNTA	PGGT1B	CTSK	HSD17B2
HSD17B1	CTSL	CTSB	NPBWR1	FLT1	GSK3B	KDR
MET	NR1I3	DNMT3A	NR1H3	NR1H2	FKBP1A	CFTR
HTR7	HTR6	BCHE	STAT3	NOS2	TLR9	LRRK2
CCNE2	PPP1CA	PBRM1	SMARCA4	IKBKE	PTGER1	PSMB5
TRPM8	PABPC1	ERBB2	DHFR	EGFR	CXCL8	PDE7A
SAE1	TGFBR1	TRPV1	NQO2	CDC42	RAC1	SIGMAR1
HTR2B	ADRA2B	CYP2D6	F3	CHRNA7	PTPN1	SLC9A1
MKNK1	HPGD	WEE1	PTK2	MMP9	MMP1	MMP2
PDK1	AKR1B1	MAOB	AURKB	PIK3CD	PRKDC	HCK
PIK3CG	PI4KB	AURKA	EPHB4	MMP8	ITK	CDK1
HTR3A	ATR	LCK	TERT	CDK4	MCHR1	PHLPP2
ELANE	SLC1A3	RAF1	RET	MMP3	HSD17B3	AGPAT2
5HT2B	5HT2A	5HT7R	ACHA2	ACHA4	ACHB2	CP1A2
DHCR7	DHPR	DRD1	DRD3	DRD2	DRD5	FKB1A
KCNN1	KCNN2	KCNN3	MMP15	MMP16	MMP26	PDE1A

S47A1	T2R31	TBB1	TF	TY3H	TYDP2	VMAT2
ADRA1A	CHRNA4	HTR1A	PTPRCAP	ADRA1D	ADRA1B	DRD4
OPRM1	HTR5A	ADRA2A	TH	HTR1B	HTR1D	GCGR
MMP7	ALOX15	ALOX12	MAP2K1	ADAM17	PAK4	CHEK1
FCER2	MMP13	ABCB1	HDAC8	EGLN1	ALK	PSEN2
ERN1	HDAC7	SRC	PDF	PDGFRA	FLT3	HSP90AB1
ILK	ANPEP	PDGFRB	KIT	FLT4	LNPEP	ALPL
PLAA	APP	PRKCZ	FYN	CDD	CXB2	FGF1
FGF2	IL2	LEG3	LEG4	LEG8	LEG9	LYAM1
LYAM3	MAG	S28A3	SC5A4	SC5A1	SC5A2	SUIS
TYRO	VEGFA	SLC5A2	ADK	BCL2L1	SLC5A1	SLC5A4
HSD11B2	GBA	SLC29A1	UPP1	PTAFR	SLC28A2	CSNK2A1
GLB1	TOP1	DYRK1A	DYRK2	FUCA1	IGFBP3	P2RY12
IMPDH2	UGCG	GAA	GBA2	EED	NQO1	RBBP4
RBBP7	SUZ12	PER2	EDNRA	MAP3K20	TGFBR2	TRPV4
TRPA1	F10	PDE4B	VHL	SCN9A	BRD4	CREBBP
BACE1	THRB	ABCC9	OPRL1	NTRK1	CHRM4	CHRM5
CHRM2	CHRM3	KIF11	YES1	CCR1	HIF1A	PKM
IL1B	FGFR1	PARP1	PDE9A	NR3C2	JAK3	KCNE1
EPHX2	JAK2	TLR7	TK1	JAK1	PDE1B	TBB5
MTNR1A	MTNR1B	TBXAS1	CYP1B1	CYP1A1	RPS6KA3	CCNC
CDK8	PGK1	PARP2	IKBKB	PLK3	PLK2	TRPC6
ROCK2	PKN2	TTK	SIRT2	TUBB1	LYN	TEK
CHEK2	GPR139	GCK	CDC25B	PPIA	GRIA1	ABCG2
CLK4	BCAT2	NAAA	CFD	KDM5B	MARK1	GRK5
PDE3A	TF65	UB2D3	F2	PRSS1	CTRC	POLB
ADH1A	XPO1	SRD5A1	MPO	ADH1C	CP1A1	CP1B1
NR0B1	PDE4A	PDE4D	PDE4C	PIP4K2C	CA12	CA9
GPR55	AHR	TACR1	BRD2	BAZ2B	BRD3	BAZ2A
TNFRSF1A	MAPKAPK	NOS1	NOS3	GPBAR1	CHRNB1	CHRNA3
MAOA	CYP2C9	CD38	BACE2	PDE3B	ERBB4	CYP2C19
CECR2	BDKRB2	NAMPT	SLC16A1	GRM4	CSF1R	CA14
QPCT	A4	ABC3A	ACES	ACH10	ACHA9	ACRO
ADA17	ADAM9	AK1BA	AK1C2	AK1C4	AK1C3	ALDR
AMPN	AOC3	AOFA	AOFB	AREG	BMP1	CAH14
CAH12	CAH4	CAH5A	CAH5B	CAH1	CAH6	CAH2
CAH7	CAH9	CASPA	CCR2	CHLE	CLK1	CP24A
CP2C9	CP3A4	CTBP2	CTDS1	DHB2	DHB1	ERCC5
ESR2	FACE2	FNTB	FOS	G6PC	GBRA2	GBRB2
GBRG2	GP183	GRIK2	GRIK1	GRP3	HCAR2	HDA11
HDAC2	HDAC3	HDAC4	HDAC1	HDAC6	HKDC1	HPSE
JUN	KEAP1	KLK14	KLF5	KLK5	KS6B2	LGUL
MCHR2	MDR1	MEP1B	MIF	MITF	MMP12	MOT4
MTR1A	MTR1B	MYLK	NEMO	NFKB1	NF2L2	NLRP3
NOD2	NPBW1	NR1H4	PABP1	PE2R2	PE2R4	PGH2
PIN4	PPBT	PPIB	PPID	PTN1	RBBP9	REV1
RPGF4	SDF1	SENP1	SENP6	SENP7	T2R14	TAU
TGM3	THAS	TNR1A	TOP2A	TS1R1	TS1R3	TTHY
XBP1	CA7	CA2	CA1	DYRK1B	CA3	CA5B
AKR1B10	CA13	CA6	CA4	CA5A	PMM2	PHOSPHO1

AKR1C3	CYP1A2	TAAR1	C1R	KAT2B	TYMP	TNKS
CTSS	CES1	CSNK1G2	CSNK1D	CES2	RPS6KA5	PLA2G1B
CDC25A	TTL	ATP12A	DCTPP1	FADS1	HMOX1	CYP51A1
SGMR2	TUBB3	GUSB	PITRM1	PLA2G7	HDAC5	EPHA3
HSP90B1	COMT	5HT1E	5HT5A	ADA2B	ADA2A	CP2D6
CXCR3	PLEC	HRH1	HTR1E	HRH4	DPP4	DPP8
DPP9	TRPV3	SLC18A3	FAP	ACKR3	SLC6A9	PNMT
CHRNE	MTAP	KDM1A	H1F0	DPP7	SLC18A2	XIAP
BIRC2	APEX1	EZH2	MPIP2	PTN6	PTPN6	PTPN11
PTPRC	MPI	CDC25C	EIF4H	EIF2AK3	PTGER3	CDK9
DUT	PREP	AGTR1	STK3	STK26	BRS3	1433S
ABCC8	ACPM	ADCY1	AGAL	ALR	B2LA1	BKRB2
CAH13	CAN1	CBR1	CIA30	CISD1	DCOR	DUS3
ERCC1	ERR3	FOXO1	GLR	HDA10	HDAC9	IMDH1
IMDH2	KCC2A	LOX12	LOX15	LSHR	LYAM2	MAP2
MCL1	MPRI	MYOC	NDUA1	NDUA2	NDUA4	NDUA3
NDUA5	NDUA6	NDUA7	NDUA8	NDUA9	NDUAA	NDUAB
NDUAC	NDUAD	NDUB1	NDUB2	NDUB3	NDUB4	NDUB5
NDUB6	NDUB7	NDUB8	NDUB9	NDUBA	NDUBB	NDUC1
NDUC2	NDUF3	NDUF2	NDUF4	NDUS1	NDUS2	NDUS3
NDUS4	NDUS5	NDUS7	NDUS6	NDUS8	NDUV1	NDUV2
NDUV3	NU1M	NU2M	NU3M	NU4LM	NU4M	NU5M
NU6M	NUA4L	PACR	PASK	PD2R	PDK3	PDK4
PE2R1	PERM	PGH1	PTN7	SC6A5	SPRE	ST17B
STAT6	SYYC	TRPM5	XDH	XPF	TYR	ALDH2
DNM1	THRA	GLI2	RPS6KB1	CCNE1	GRK2	ESRRA
ESRRB	INSR	EPHA2	EPHB2	EPHA5	EPHA4	EPHA8
EPHA7	EPHB3	EPHB1	EPHA1	AKT2	HPGDS	ATP4B
TNF	BRD9	BCL2	TRAP1	TBXA2R	MELK	P2RX1
PLCG1	CCR5	CCR8	CTSH	RXRB	RXRG	RXRA
MME	NEK1	CTSD	GABRG2	CASP3	LTB4R	ECE1
TNKS2	IMPDH1	CASR	ATM	AL1A2	AL1B1	AMYP
B4GT1	CD69	ERAP1	HEXA	HEXB	IL6	LEG1
LEG7	MGA	MGMT	NMUR2	P2Y14	PDCD4	PDIA1
RASH	S28A2	S29A1	SC5AB	TYDP1	SQLE	AMY1A
SERPINE1	PTPN2	CSNK2A2	PBK	AADAT	CHUK	PNP
PRMT6	PRMT8	PRMT1	IRAK4	ROCK1	PKN1	PRKD1
ADCY5	P2RX4	PIM3	HTR1F	ERAP2	PDPK1	LIMK1
FPR2	RPS6KA2	MST1R	PTGER2	BHMT1	CELA1	CP26B
CP26A	CSK22	CYC	DNLI1	DOPO	ERR2	FOLH1
FTO	GABT	GLRA3	HAOX1	HCAR3	HIF1N	HNF4A
IBP1	IBP2	IBP4	IBP5	IBP6	KDM2A	KDM3A
KDM4A	KDM4D	KDM4E	KDM5C	KDM4C	KKCC2	MYG
NALD2	NEUR3	NR4A2	NR4A1	OXDA	OXDD	P4HA1
P4HTM	PAR15	PAR16	QCR7	RARG	RFA1	S22A6
S22A8	S5A2	SSDH	TPMT	UBP4	UBP5	XCT
SRD5A2	DAO	AKR1C2	AKR1C1	LDHA	LDHB	1433G
CH10	CH60	CP2C8	CP2A6	DAPK2	ELAV1	ELAV3
NEK6	NOX4	NSD2	PAI1	PPBI	PPBN	PSDE
Q9UM81	SIAT1	SYUA	DUSP1	ADA2C	AGTR2	HCN4

MRP1	OX1R	OX2R	CHRNB4	ABCC1	SLC47A1	OPRD1
OPRK1	BTK	SLC10A2	MKNK2	PLA2G10	CASP1	TNNC1
FBP1	CAPN1	CASP7	EDNRB	ACPP	NFKBIA	RELA
CYP3A4	CASP8	LGMN	FDFT1	GABRA5	HRH3	TYK2
CMA1	IDO1	ACLY	CXCR1	PRKACA	CPT1A	CCR9
HPD	NRP1	CETP	CALCA	GZMB	TACR3	GRIN1
ADH5	ALKBH3	PLAP	AKT1	LIPC	UTS2R	LIPG
GLO1	IKBKG	SIRT1	HTR4	TACR2	TSHR	CD22
G3P	TNNI3	TNNT2	HSPA5	SLC2A1	HK2	HK1
HSPA8	NADK	SLC5A11	CCND3	CHK1	G6PT1	KCMA1
KCNB1	NOX1	NAT1	ALPG	ADCYA	ANDR	AT12A
CBG	CP17A	CP19A	DHB7	G6PD	GBRA1	
GPBAR	GPER1	LICH	MRP4	NMDE2	NMDE3	
NMDZ1	NPCL1	NTCP	S22A1	S5A1	SIA4A	
UGT2B7	SERPINA6	PPM1B	PPP1CC	MAPK3	PPP5C	
HSD17B7	MAP3K5	MAP3K11	GABBR2	MAPK9	PPARD	
RHOA	PRMT3	PAM	SO4C1	NMDE4	GBRD	
