# Supplementary material for: Potential mechanism of Luoshi Neiyi prescription in endometriosis based on serum pharmacochemistry and network pharmacology
Source: Front Pharmacol. 2024 Jul 29;15:1395160. doi: 10.3389/fphar.2024.1395160 (PMC11317381; doi:10.3389/fphar.2024.1395160)
Supplement: Supplementary file 5 [file DataSheet1.PDF]

Table S1: 185 components in LSNYP		
Trigonelline	Licoflavone A	Tetrahydrocolumbamine
Quercetin	Ethyl ferulate	1-(4-nonylphenoxy)-2-propanol
Rutin	Cryptopine	Protocatechuic acid
Salsoline	Saurufuran A	Mandelic amide-β-gentiobioside
Aesculitannin B	Hispidulin	N-Methylcoclaurine-7-O-β-D-glucopyranoside
Salviaflaside	Jatrorrhizine	n-Butylidenephthalide
Thaliporphine	Sinapic acid	Salvianolic acid H/L/I
Lindeneol A	Isohemiphloin	p-Hydroxybenzoic acid
Tanshinol A	Danshenxinkun D	Apigenin 6-C-arabinoside 8-C-glucoside
Corypalmine	Forsythoside D	Chuanxiongnolide R2
Norisoboldine	Norbracteoline	7-Hydroxycoumarin
Typhaneoside	Danshensu	Isorhamnetin-3-O-neohesperidoside
Nikoenoside	Senkyunolide K	Quercetin-3-O-β-D-glucoside
Amygdalin	Senkyunolide J	Mandelic acid-β-gentiobioside
Tanshinone VI	Bulbocapnine	1-O-Caffeoyl-β-D-glucopyranoside
Boldine	Senkyunolide P	4-O-Acetyl-caffeic acid
Tanshindiol B	Peimisine	8-O-Acetyl shanzhiside methyl ester
Hyperoside	Ferulic acid	Kaempferol-3-O-rutinoside
Luteolin	Karakoramine	Vitexin -4''-O-glucoside
Senkyunolide N	Picrasidine I	6,7-Dihydroxy-2-(2-phenylethyl) chromone
Tanshinol B	Norglaucine	6-Hydroxykaempferol-3-O-glucoside
Caffeic acid	Stepholidine	Kaempferol 3-O-neohesperidoside
Isoboldine	Kaempferol	Isorhamnetin-3-glucoside
Isoquercitrin	Lysergol	Ethyl 4-methoxycinnamate
Vanillin	Linderane	3,5-O-Dicaffeoylquinic acid
Hernangerine	Salannin	Sipeimine-3-β-D-glucoside
Macrophylloside A	Tanshinone IIA	Neocryptotanshinone
Syringic acid	E-Ligustilide	Protocatechualdehyde
Tanshindiol C	Rotundine	Dihydrotanshinone I
Verbascoside	Perlolyrine	1,3-O-Dicaffeoylquinic acid
Corydine	Myristicin	Salvianolic acid D/G
Byzantionoside B	Lirioferine	Olivil-4''-O-β-D-glucopyranoside
Senkyunolide G	Peiminine	Trifolirhizin 6'-monoacertate
Cynaroside	Neoeriocitrin	Salvianolic acid H/L/I
Leonticine	Lindestrene	Isocryptotanshinone
Berberine	Frumariline	Tetrahydropalmatine
Linalyl rutinoside	Trijuganone C	3-Butylidene-7-hydroxyphthalide

Peimine	Tanshinoldehyde	Vitexin-2"-O-rhamnoside
Zhebeirine	Colchicine	β,β-Dimethylacryl-shikonin
Salvianolic acid C	Kuraridinol	Dihydroberberine
Salvianolic acid A	Hupehensine	13-Methyl-dehydrocorydalmine
Wogonin	Palmatine	11-hydroxy-3,21-diacetate-20-progesterone
Danshenol B	Columbamine	Methyl dihydrotanshinonate
Senkyunolide B	Moupinamide	Chlorogenic acid
Dihydronitidine	Corycavine	Dehydrocostuslactone
Apigenin	Adoxosidic acid	Salvianolic acid G
Evocarpine	Ganoderic acid A	5α,14α-Cevanine-3β,2-dihydroxy-6-one
Terminalic acid	Lindenenol	1,2-Dihydrotanshinone IIA
Chuanbeinone	Cryptotanshinone	Hydroxylinderstrenolide
Andropanolide	Senkyunolide A	Ganoderenic acid A
Yuanamide	Picrasinoside A	Lithospermic acid
Decarine	Zhebeininoside	Salvianolic acid H/L/I
Naringenin	Allocryptopine	Neochlorogenic acid
Linderalactone	Phloroglucinol	Apigenin 7-glucoside
Procyanidin B2	Corydaline	17β-Hydroxy--2-oxa-5α-androstan-3-one
Saurufuran B	Sinpeinine A	1,2-Dihexyloxybenzene
Acetoxypinoresinol	Dihydropalmatine	Tetrahydroberberine
Mulberrofuran A	Danshenxinkun A	Lsolinderalactone
Ebeiedine	6-Hydroxypurine	9-Carbonyl-10E,12Z-octadecadienoic acid
Shizukanolide A	Dihydrochelerythrine	ent-16β,17-Dihydroxy-19-kauranoic acid
Senkyunolide F	Dehydrocorydaline	2α,3β,19α-Trihydroxyursolic acid
Deoxyschizandrin	Dihydrosanguinarine	
