# Supplementary material for: Potential mechanism of Luoshi Neiyi prescription in endometriosis based on serum pharmacochemistry and network pharmacology
Source: Front Pharmacol. 2024 Jul 29;15:1395160. doi: 10.3389/fphar.2024.1395160 (PMC11317381; doi:10.3389/fphar.2024.1395160)
Supplement: Supplementary file 6 [file DataSheet5.PDF]

Table S5: 182 key targets of LSNYP in EMs treatment

ADA	CNR1	NR5A2	PPARG	S1PR1
STS	AR	PTGS2	SLC6A4	VDR
MAPK14	IGF1R	IL6ST	PRKCB	ESR1
SHBG	ABL1	BRAF	MAPK1	MTOR
PIK3CA	CYP24A1	PLK1	NR3C1	EPHX1
MMP14	ALOX5	CCND1	AXL	PTGES
P2RX3	RBP4	TLR4	RORC	CYP19A1
PTGS1	PGR	CXCR2	CYP17A1	HSD17B2
HSD17B1	CTSB	FLT1	GSK3B	KDR
MET	DNMT3A	NR1H2	STAT3	NOS2
ERBB2	EGFR	CXCL8	TGFBR1	TRPV1
CDC42	F3	HPGD	PTK2	MMP9
MMP1	MMP2	AKR1B1	AURKB	AURKA
CDK1	TERT	ELANE	RAF1	MMP3
DRD2	TF	OPRM1	TH	MMP7
ALOX15	ADAM17	PAK4	MMP13	ERN1
SRC	FGF1	FGF2	IL2	VEGFA
BCL2L1	GLB1	IGFBP3	UGCG	EED
EDNRA	NTRK1	CCR1	HIF1A	IL1B
FGFR1	JAK1	CYP1B1	CYP1A1	ROCK2
TEK	ABCG2	F2	SRD5A1	MPO
AHR	TACR1	TNFRSF1A	NOS3	CYP2C19
BDKRB2	CSF1R	CCR2	ERCC5	ESR2
FOS	HDAC2	HDAC1	HPSE	JUN
MIF	MMP12	NFKB1	NLRP3	AKR1C3
TYMP	CDC25A	HMOX1	COMT	CXCR3
SLC18A3	ACKR3	BIRC2	APEX1	EZH2
EIF2AK3	AGTR1	CBR1	FOXO1	STAT6
XDH	ALDH2	RPS6KB1	TNF	BCL2
CCR5	CCR8	MME	CTSD	CD69
IL6	SERPINE1	CHUK	ROCK1	LIMK1
MST1R	FTO	NR4A1	AKR1C1	LDHA
CASP1	NFKBIA	RELA	CYP3A4	CASP8
TYK2	IDO1	CXCR1	CCR9	NRP1
CETP	CALCA	AKT1	LIPC	SIRT1
NAT1	GPER1	SERPINA6	MAPK3	HSD17B7
MAPK9	RHOA			
